# Phagocytosis-like cell engulfment by a planctomycete bacterium

**DOI:** 10.1038/s41467-019-13499-2

**Published:** 2019-12-11

**Authors:** Takashi Shiratori, Shigekatsu Suzuki, Yukako Kakizawa, Ken-ichiro Ishida

**Affiliations:** 1 Faculty of Life and Environmental Sciences, University of Tsukuba, Tsukuba, Ibaraki 305-0053 Japan; 2 Japan Agency for Marine-Earth Science and Technology (JAMSTEC), Yokosuka, Kanagawa 237-0061 Japan; 3 National Institute for Environmental Studies, Tsukuba, Ibaraki 305-0053 Japan

**Keywords:** Cell biology, Bacterial evolution, Bacterial genomics, Cellular microbiology

## Abstract

Phagocytosis is a key eukaryotic feature, conserved from unicellular protists to animals, that enabled eukaryotes to feed on other organisms. It could also be a driving force behind endosymbiosis, a process by which α-proteobacteria and cyanobacteria evolved into mitochondria and plastids, respectively. Here we describe a planctomycete bacterium, ‘*Candidatus* Uab amorphum’, which is able to engulf other bacteria and small eukaryotic cells through a phagocytosis-like mechanism. Observations via light and electron microscopy suggest that this bacterium digests prey cells in specific compartments. With the possible exception of a gene encoding an actin-like protein, analysis of the ‘*Ca*. Uab amorphum’ genomic sequence does not reveal any genes homologous to eukaryotic phagocytosis genes, suggesting that cell engulfment in this microorganism is probably not homologous to eukaryotic phagocytosis. The discovery of this “phagotrophic” bacterium expands our understanding of the cellular complexity of prokaryotes, and may be relevant to the origin of eukaryotic cells.

## Abstract (web summary)

Phagocytosis is a typically eukaryotic feature that could be behind the origin of eukaryotic cells. Here, the authors describe a bacterium that can engulf other bacteria and small eukaryotic cells through a phagocytosis-like mechanism.

## Introduction

There are significant differences between eukaryotes and prokaryotes in terms of cellular and genomic complexity^1,2^. During the emergence of ancestral eukaryotes (eukaryogenesis), cells increased in size and developed an endomembrane system, a nucleus and other membrane-bounded organelles. They also developed actin- and tubulin-based cytoskeletons, which facilitated uptake of large particles from the environment (phagocytosis)^3^. Phagocytosis not only supports efficient nutrient acquisition via consumption of other organisms (phagotrophy), but is also considered by some eukaryogenesis theories to be involved in the acquisition of the mitochondrion^4–6^. However, it remains unclear how the ancestral eukaryote acquired the phagocytosis ability since no known present organism exhibits a primitive phagocytosis.

Recent metagenomic studies have identified an archaeal group, Asgard, most closely related to Eukarya^7,8^. Asgard archaea possess genes that are homologous to eukaryotic genes involved in cytoskeleton formation, vesicle/membrane trafficking or remodelling and phagocytosis^7,8^. Recent cultivation and physiological studies of an Asgard archaeon, *‘Candidatus* Prometheoarchaeum syntrophicum’, have shown that this organism is syntrophic with bacteria and occasionally produces long, branching protrusions^9^. However, it has not been reported that *‘Ca*. Prometheoarchaeum syntrophicum’ displays any phagocytic-like behaviour.

Here, we describe a planctomycete bacterium, ‘*Candidatus* Uab amorphum’ (named after a hungry giant in Palauan mythology, called Uab) that exhibits several intriguing eukaryotic-like features, including the ability to engulf other microorganisms (bacteria and eukaryotes) through a phagocytosis-like process.

## Results

### Characteristics of ‘*Ca*. Uab amorphum’

**Fig. 1:**
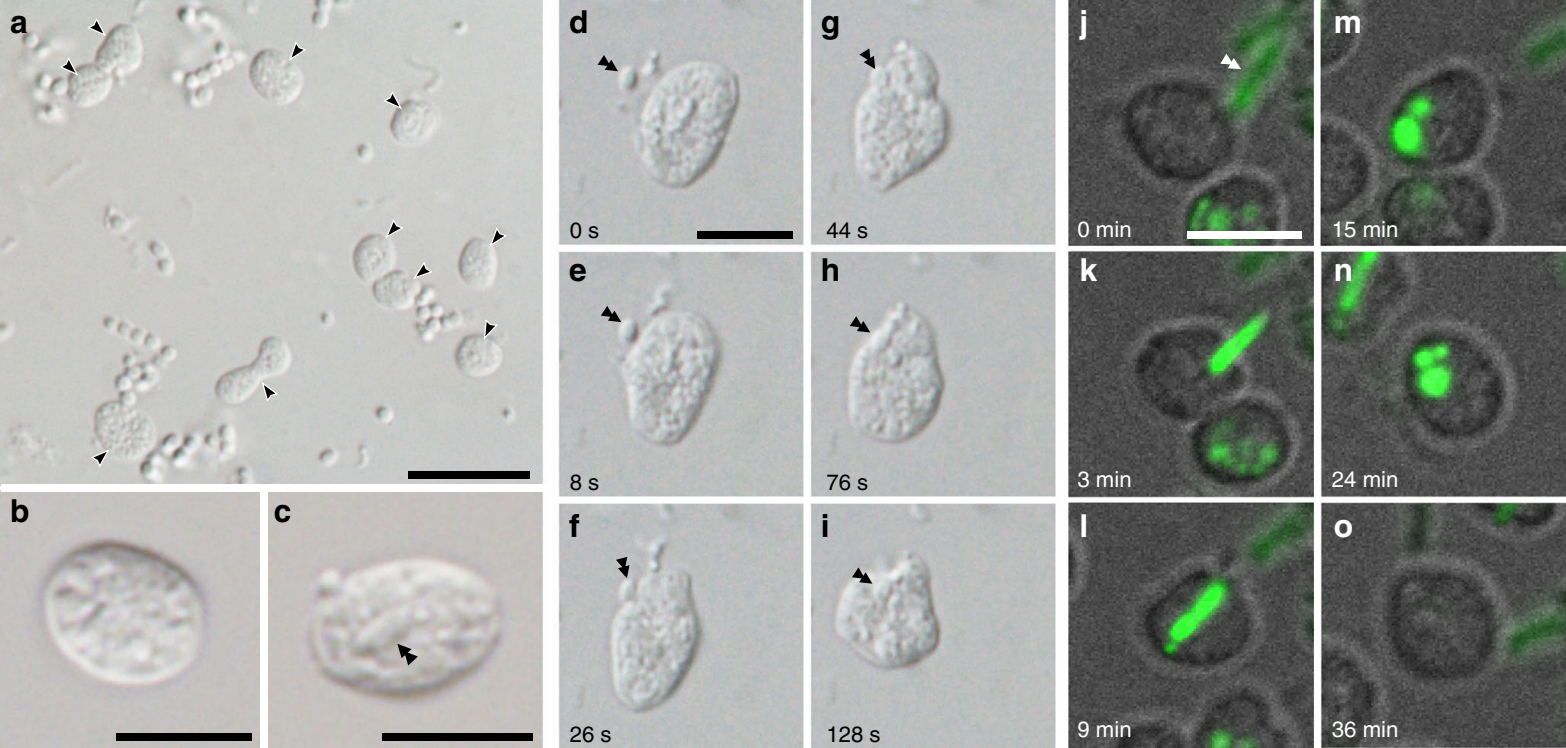
**Light and fluorescent micrographs of ‘*Candidatus*** **Uab amorphum’.** **a** Cells of ‘*Ca*. Uab amorphum’ (arrowheads) in the xenic culture. **b**, **c** A cell of ‘*Ca*. Uab amorphum’. Double arrowheads indicate an engulfed bacterium. **d**–**i** Selected images of time-lapse video showing prey engulfment process of ‘*Ca*. Uab amorphum’. **j**–**o** Selected images of confocal fluorescent time-lapse video showing prey engulfment and digestion process of ‘*Ca*. Uab amorphum’. Green fluorescence indicates AcGFP1-labelled *Escherichia coli*. Scale bars, 10 μm (**a**) and 5 μm (**b**–**o**).

*‘Ca*. Uab amorphum’ was discovered from a surface seawater sample collected at the Republic of Palau. It was maintained as an enriched or as a monoxenic culture with *Alteromonas macleodii* (NBRC 102226). Our attempts to establish axenic cultures were unsuccessful. We found that ‘*Ca*. Uab amorphum’ has a flat, round or oval cell shape that is ~4–5 μm in diameter (Fig. 1a–c). However, cells occasionally reached 10 μm in diameter when cultures included many co-cultured bacterial cells (Supplementary Fig. 1a). ‘*Ca*. Uab amorphum’ moved on solid substrates at approximately 8–16 μm/min while frequently changing cell shape (Supplementary Movie 1). In addition, ‘*Ca*. Uab amorphum’ engulfed other bacteria in cultures (Fig. 1d, e, Supplementary Movie 2); the species description is included in the ‘Methods’ section.

Amplification of ribosomal RNA (rRNA) using PCR and fluorescent in situ hybridization (FISH) with specific oligonucleotide probes for ‘*Ca*. Uab amorphum’ revealed that this organism was not a eukaryotic amoeba, but a bacterium whose 16 S rRNA gene sequence similarity with the closest known species was only 79% (Supplementary Fig. 2, Supplementary Table 1). Genome sequencing of the monoxenic culture produced a circular genome that encoded the 16 S rRNA of ‘*Ca*. Uab amorphum’; the remaining sequences were small portions of the *A. macleodii* genomes (Supplementary Table 2). The ‘*Ca*. Uab amorphum’ genome was AT rich, 9.5 Mb in size and encoded 6660 protein-coding genes without plasmids (Supplementary Table 3).

**Fig. 2:**
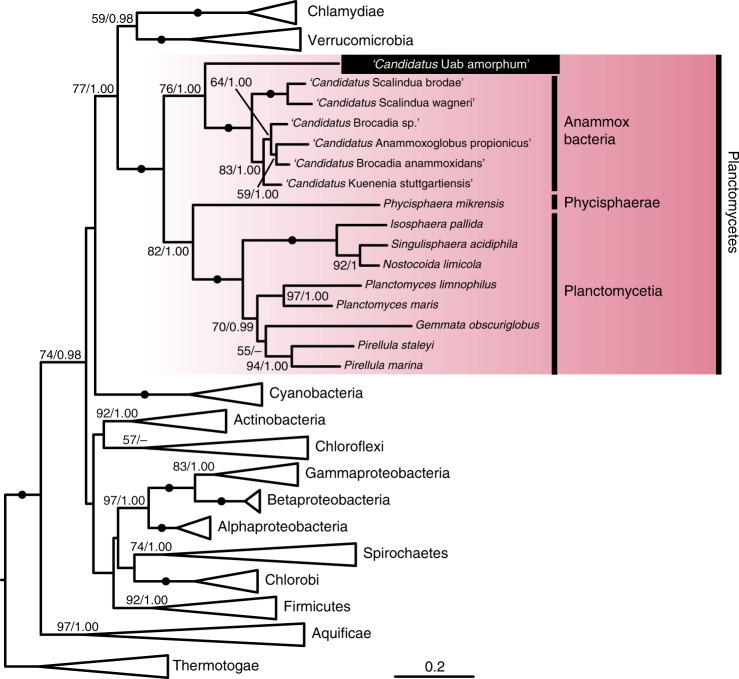
**Maximum likelihood tree of ‘*Ca*. Uab amorphum’ and other bacterial 16S rRNA gene sequences.** Left and right values on nodes indicate bootstrap value and Bayesian posterior probability, respectively. Scale bar indicates nucleotide substitution rate per site.

To identify the phylogenetic position of ‘*Ca*. Uab amorphum’, we performed molecular phylogenetic analyses with two datasets, one using 16S rRNA gene sequences and the other using 171 proteins. Both analyses indicated that ‘*Ca*. Uab amorphum’ belongs to the bacterial phylum Planctomycetes (Fig. 2, Supplementary Fig. 3). ‘*Ca*. Uab amorphum’ was genetically separated from three subgroups of Planctomycetes: Planctomycetia, Phycisphaerae and anammox (anaerobic ammonium oxidation) bacteria^10^, with anammox bacteria being its closest relative. We found that various environmental rRNA gene sequences formed a clade with ‘*Ca*. Uab amorphum’ (Supplementary Fig. 4). This “Uab clade” included sequences derived from freshwater, hypersaline and marine environments, as well as the bovine rumen. The similarity between ‘*Ca*. Uab amorphum’ and these environmental sequences varied between 79.4% (KC604713) and 98.4% (KU631341). These data suggest that relatives of ‘*Ca*. Uab amorphum’ live in various environments and have high genetic diversity. We also screened 16S rRNA data from the Tara Oceans expedition^11^ and found sequences related to the “Uab clade” in datasets derived from stations in the North and South Atlantic Oceans, the Indian Ocean, the North and South Pacific Ocean, the Red Sea and the Southern Ocean (Supplementary Fig. 5).

**Fig. 3:**
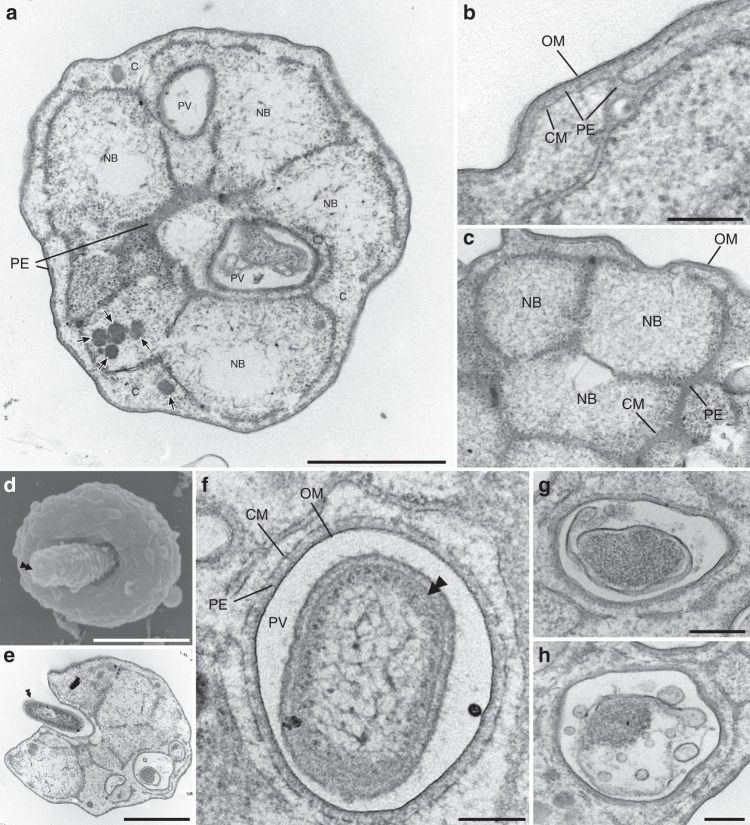
**Ultrastructure of ‘*Candidatus*** **Uab amorphum’.** **a** Transmission electron micrograph of ‘*Ca*. Uab amorphum’ showing cytoplasm (C), cytoplasmic membrane (CM), nuclear body (NB), outer membrane (OM), periplasm (PE) and phagosome-like vacuole (PV). Arrows indicate dense granules that are probably glycogen granules. **b**, **c** High-magnification transmission electron micrograph showing the membrane structures. Cytoplasmic membrane (CM), nuclear body (NB), outer membrane (OM) and periplasm (PE). **d** Scanning electron micrograph of a ‘*Ca*. Uab amorphum’ that is about to engulf a prey bacterium (double arrowhead). **e** Transmission electron micrograph of a ‘*Ca*. Uab amorphum’ that is about to engulf a bacterium (double arrowhead). **f** Ultrastructure of PV that is surrounded by outer membrane (OM), periplasm (PE) and cytoplasmic membrane (CM). Double arrowhead indicates engulfed prey bacterium **g**, **h** that disrupted prey bacteria in PVs. Scale bars, 1 μm (**a**, **d**, **e**), 500 nm (**b**, **h**) and 200 nm (**c**, **f**, **g**).

Transmission electron microscopy (TEM) demonstrated that ‘*Ca*. Uab amorphum’ has two membrane-bounded regions: an electron-dense ribosome-free region and a ribosome-containing region (Fig. 3a–c). The ribosome-free region located immediately beneath the outermost membrane and was highly invaginated into the ribosome-containing region, which showed a reticulate pattern in thin-section micrographs. The ribosome-containing region had multiple nuclear bodies that appeared to be separated by the invaginated ribosome-free region. This cellular structure consisting of ribosome-free and ribosome-containing regions with complex invaginations is typical of some planctomycete bacteria such as *Gemmata obscuriglobus*^12^. On the basis of electron microscopic observation and distribution of RNA^13^, it has been previously proposed that the outermost membrane surrounding the ribosome-free region is the cytoplasmic membrane, and the membrane surrounding the ribosome-containing region is an intracytoplasmic membrane. However, recent genomic and cell biological studies indicate that the cellular structure of planctomycetes resembles that of typical Gram-negative bacteria, with the outermost and inner membranes being equivalent to the outer membrane and cytoplasmic membrane, and the ribosome-free and ribosome-containing regions corresponding to the periplasm and cytoplasm, respectively^14,15^. We found that the genome of ‘*Ca*. Uab amorphum’ includes genes encoding proteins similar to Gram-negative bacterial type II secretion systems (T2SS) (Supplementary Table 4), which would be consistent with a Gram-negative bacterial cell plan.

The genome of ‘*Ca*. Uab amorphum’ possesses most of the genes required for peptidoglycan biosynthesis (Supplementary Table 5), but the presence and properties of a peptidoglycan layer remain untested. Considering its cell flexibility, this organism is unlikely to have a rigid peptidoglycan layer. Synthesis and degradation of the peptidoglycan layer are known to be required for engulfment of the daughter cell during spore formation in other bacterial species^16,17^.

In comparison with other planctomycetes, ‘*Ca*. Uab amorphum’ exhibited a relatively large and gene-rich genome (Supplementary Table 3), with many genes involved in signal transduction (Supplementary Fig. 6). In addition, we identified 1515 genes of unknown function, and 1823 genes (27.6% of all genes) potentially derived from horizontal gene transfer (HGT) (Supplementary Table 6). Most of these HGT candidates (1778 genes) were apparently derived from bacteria. We detected only two genes potentially derived from eukaryotes, encoding proteins similar to actin (UABAM_01738) and acyloxyacyl hydrolase (AOAH; UABAM_05422), respectively. ‘*Ca*. Uab amorphum’ apparently lacks other eukaryotic-derived genes previously reported in Planctomycetes, such as genes associated with sterol biosynthesis, membrane coat protein, integrin and the inter-alpha-trypsin inhibitor^18–20^. We searched for homologues of eukaryote signature proteins (ESPs)^21^ and found 27 ESP candidates (Supplementary Table 7). Most of these candidates were more similar to other bacterial proteins than to eukaryotic proteins, with the exception of an actin-like protein and four WD40 repeat-containing proteins (Supplementary Table 7). However, the actin-like protein appears to be more closely related to certain archaeal actins than to eukaryotic actins (see the section ‘Fibrous structures and an actin-like protein’).

### Phagocytosis-like cell engulfment

The most notable feature of ‘*Ca*. Uab amorphum’ is its ability to engulf other organisms (prey), including Gram-negative bacteria (*Alteromonas macleodii* and *Escherichia coli*), Gram-positive bacteria (*Lactobacillus farciminis* and *Staphylococcus condimenti*) and a picoeukaryotic alga (*Bathycoccus prasinos*) (Supplementary Table 8, Supplementary Fig. 7). However, it was not able to engulf a marine yeast *Debaryomyces hansenii*, possibly due to its relatively large cell size (ca. 5 μm in diameter, Supplementary Movie 3). Light and fluorescent microscopy showed that prey cells were taken up by surface invagination of the ‘*Ca*. Uab amorphum’ cell. In the presence of sodium azide (which inhibits the ATP hydrolase activity of F-ATPase), cells did not show any locomotion or cell engulfment, which suggest that these may be energy-dependent processes (Supplementary Fig. 1b, c). Feeding experiments by using an AcGFP1-labelled *E. coli* showed that the rod-shaped signals were separated into several globules (Fig. 1j–o, Supplementary Video 4) that disappeared over time and did not diffuse into the cell. We detected few acidic and reactive oxygen species (ROS) signals in cells of ‘*Ca*. Uab amorphum’ (Supplementary Fig. 1d–i). Electron microscopy analyses showed invagination of the ‘*Ca*. Uab amorphum’ cell surface around prey cells (Fig. 3d, e). Consistent with the prediction that both outer and cytoplasmic membranes invaginate during prey engulf, the resulting compartment enclosing the prey cell (phagosome-like vacuole, PV) appeared to be surrounded by two membranes, i.e. the inner membrane of the PV would be the putative Gram-negative outer membrane, and the outer membrane of the PV would be the putative cytoplasmic membrane (Fig. 3f). Most prey cells in PVs were found to be disrupted to some extent (Fig. 3g, h), which suggests that digestion may occur in PVs. Some PVs exhibited narrow ducts that appeared to be connected to the outside of the cell, or to another PV (Supplementary Fig. 8a, b). However, serial sectional observation showed that some PVs apparently lacked such ducts. It is therefore possible that ducts may disappear over time, and that PVs may eventually close (Supplementary Fig. 8c–k).

**Fig. 4:**
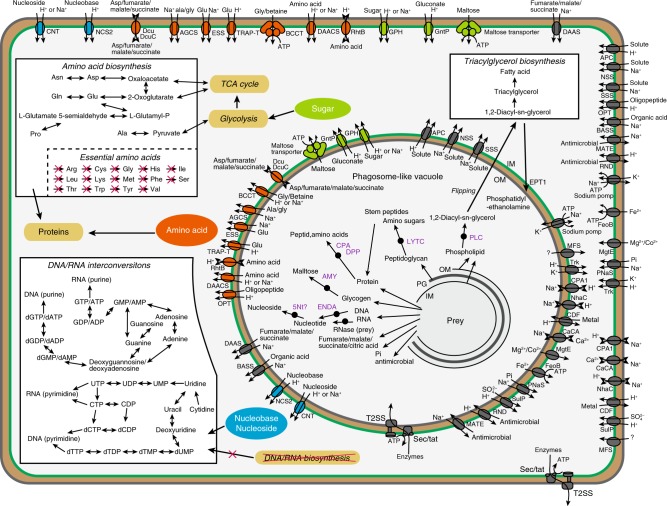
**Putative transporters, enzymes and pathways of ‘*Candidatus*** **Uab amorphum’.** Arrows indicate pathways that are present. Pathways that are absent are crossed out. Purple enzymes are predicted to be secreted in PV or localized at the outer membrane and involve to degradation of prey. Green line indicates the cytoplasmic membrane. Grey line indicates the outer membrane. Brown layers indicate periplasm. Blue circles indicate nucleoside and nucleotide transporters. Green circles indicate sugar transporters. Orange circles indicate peptide and amino acid transporters.

The ‘*Ca*. Uab amorphum’ genome includes genes encoding putative digestive enzymes, which might degrade prey components. We detected 179 putative peptidases (Supplementary Data 1), and some digestive proteins that may be secreted or localized at the outer membrane (Fig. 4, Supplementary Table 9). Genes encoding α-amylase, carboxypeptidase and deoxyribonuclease I may have been acquired via HGT from Bdellovibrionales or Myxococcales, which are known to digest bacteria outside of the cells^22,23^ (Supplementary Fig. 9a–c). In mammals, AOAH (one of the proteins that might have been obtained by HGT from eukaryotes, Supplementary Fig. 9d) degrades and inactivates lipopolysaccharides of the bacterial outer membrane^24^. Therefore, it is possible that the putative AOAH counterpart in ‘*Ca*. Uab amorphum’ could potentially be also involved in degradation of bacterial lipopolysaccharides.

In Gram-negative bacteria, the Sec/Tat pathway transports proteins from the cytoplasm to the periplasm, and the T2SS transports proteins from the periplasm to the outside of cells^25^. We found genes encoding most of the components of the Sec/Tat pathway and T2SS in the ‘*Ca*. Uab amorphum’ genome (Supplementary Table 4). We speculate that these secretion systems may be used to secrete digestive enzymes into the PV. Other genes, encoding putative transporters of amino acids, nucleosides and sugars (Fig. 4), might be involved in the utilization of prey components.

De novo phospholipid synthesis may be important for formation and repair of PV membranes in *‘Ca*. Uab amorphum’. Phosphatidylethanolamine (PE) is one of the main phospholipids in bacterial membranes and is synthesized from phosphatidylserine (PS) by phosphatidylserine decarboxylases (PSD) in bacteria^26^. In addition to a PSD gene candidate, we found that ‘*Ca*. Uab amorphum’ has two genes potentially encoding ethanolamine phosphotransferase (EPT1; UABAM_02218 and UABAM_03998), which is also found in a few myxobacteria and eukaryotes (Supplementary Fig. 10a). EPT1 synthesizes PE from 1,2-diacyl-sn-glycerol (DAG), which is the degradation product of PE and phosphatidylcholine^27^.

### Metabolic adaptation to phagotrophy

The ‘*Ca*. Uab amorphum’ genome includes many genes similar to antibiotic-resistance genes (Supplementary Table 10). Planctomycetes are known to have β-lactamase;^28^ however, we found genes encoding putative drug efflux systems, most of which are potentially derived from HGT. We speculate that these genes might confer protection against prey antibiotics that are released into the PV.

Dependence on phagotrophy can often result in starvation due to unstable food source. Therefore, certain predatory bacteria have developed specific resting forms to resist starvation^23,29^. However, we were unable to observe any ‘*Ca*. Uab amorphum’ resting forms via microscopy.

‘*Ca*. Uab amorphum’ possesses genes potentially encoding enzymes for the synthesis of triacylglycerol (TAG) from phospholipid via DAG (Supplementary Fig. 10b–d). Given that genes for this pathway were not detected in the genomes of non-phagotrophic relatives, we speculate that ‘*Ca*. Uab amorphum’ may use this metabolic pathway to store energy as TAG in order to counter starvation.

Phagotrophy can drive degenerative evolution in metabolism. Our genomic analysis suggested that ‘*Ca*. Uab amorphum’ may be able to synthesize only six amino acids de novo (Fig. 4, Supplementary Data 2). The inability to synthesize some amino acids is found in animals, phagotrophic and parasitic protists and predatory bacteria that acquire nutrients from preys or hosts^30,31^. Interestingly, ‘*Ca*. Uab amorphum’ also lacked candidate genes for de novo purine and pyrimidine biosynthesis, but had genes for salvage pathways (Fig. 4, Supplementary Figs. 11 and 12). It is therefore possible that ‘*Ca*. Uab amorphum’ must acquire some amino acids and nucleotides from prey. The absence of de novo purine and pyrimidine biosynthesis pathways in some obligate parasites has previously been reported^32,33^.

### Fibrous structures and an actin-like protein

**Fig. 5:**
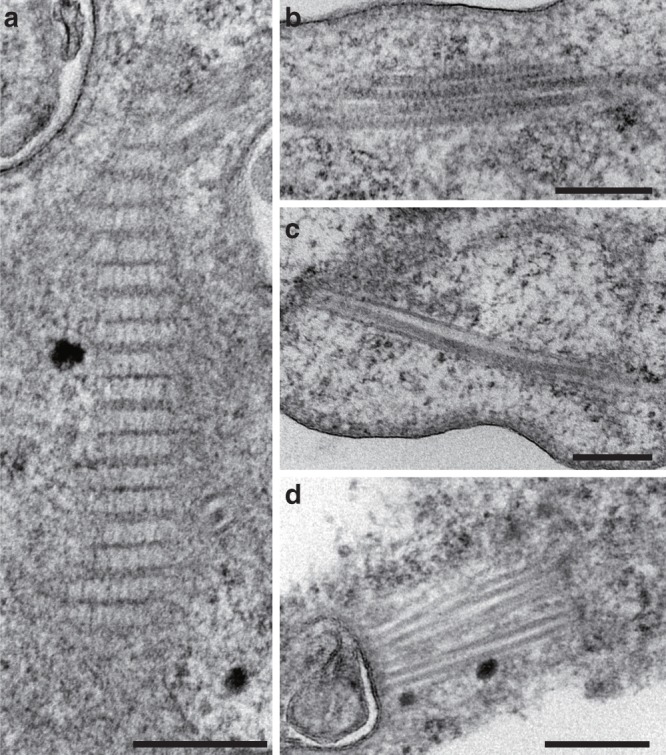
**Fibrous structures in ‘*Candidatus*** **Uab amorphum’.** **a** Large striated fibre. **b** Small striated fibre. **c** Short bundled fibre. **d** Cylindrical structures containing a linear fibre. Scale bars, 200 nm.

Actin is a eukaryote-specific protein that forms filaments by polymerization and plays crucial roles in cellular motility, cytoskeleton formation and phagocytosis^34^. TEM observation showed that ‘*Ca*. Uab amorphum’ seems to have at least four different types of fibrous structures. These include large striated fibres that occasionally reached 2 µm in length, small striated fibres, short bundled fibres and cylindrical structures that contain linear fibres (Fig. 5a–d). Such conspicuous and large fibrous structures have not been reported in other bacteria as far as we know; we therefore propose that these structures may be involved in the specific locomotion and engulfment processes displayed by ‘*Ca*. Uab amorphum’.

**Fig. 6:**
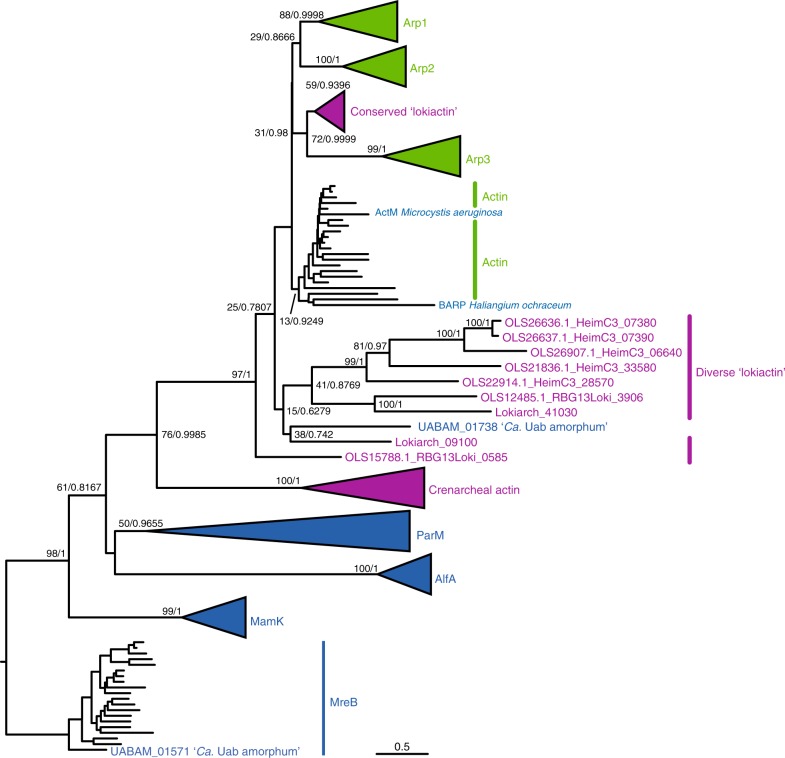
**Maximum likelihood tree of actin-like proteins.** Left and right values on nodes indicate bootstrap value and Bayesian posterior probability, respectively. Bacterial actin-related protein (BARP) of *Haliangium ochraceum* and ActM of *Microcystis aeruginosa* form a clade with eukaryotic actin. Scale bar indicates amino acid substitution rate per site.

As we previously mentioned, the ‘*Ca*. Uab amorphum’ genome includes a gene encoding an actin-like protein. Molecular phylogenetic analysis showed that this protein was distant from the bacterial actin homolog MreB and the Crenarchaeal actin, and formed a clade with eukaryotic actins, actin-related proteins (ARPs) 1–3, bacterial actin-related protein (BARP) from *Haliangium ochraceum*^35^, ActM from *Microcystis aeruginosa*^36^ and Asgard actins (Fig. 6). This analysis suggests that the Uab actin may not have been directly transferred from eukaryotes, but possibly derived from an Asgard or related archaeon. We investigated the presence of genes encoding actin-binding proteins (known to be involved in assembly, stabilisation and cross-link of actin filament^37^) and found two candidate proteins (UABAM_01722 and UABAM_04996) that are similar to Aip1 and Crn1, respectively (Supplementary Table 11). We used F-actin-binding phalloidin and an antibody specific for eukaryotic actin but failed to observe a signal on ‘*Ca*. Uab amorphum’ cells when examined by fluorescent microscopy. Therefore, it remains unclear whether the actin-like protein, or the putative actin-binding proteins, form fibrous structures and/or whether they are involved in the organism’s unique locomotion and engulfment abilities.

## Discussion

In this study, we describe the bacterium ‘*Candidatus* Uab amorphum’, which was discovered from a surface seawater sample collected at the Republic of Palau. Molecular phylogenetic analyses indicate that the organism belongs to the phylum Planctomycetes and is closely related to anammox bacteria. Planctomycetes are known to have unusual eukaryote-like features such as budding cell division, sterol synthesis and uptake of macromolecules via endocytosis-like behaviour. The endocytosis-like uptake was reported in *Gemmata obscuriglobus* as an ATP-dependent, membrane-involving process with similarities to eukaryotic endocytosis;^38^ however, a recent study reported that the macromolecules are accumulated in the periplasm^13^. Species of anammox bacteria have a unique membrane-bounded organelle called the anammoxosome that is involved in ammonium oxidation^39^. ‘*Ca*. Uab amorphum’ presents other eukaryotic-like features, such as a large and flexible cell, amoeba-like locomotion and uptake of other organisms by phagocytosis-like engulfment. In contrast to the endocytosis-like process previously reported for *G. obscuriglobus*, the phagocytosis-like engulfment observed in ‘*Ca*. Uab amorphum’ appears to involve invagination of both outer and inner membrane, and the resulting PV seems to be surrounded by a double membrane. In *G. obscuriglobus*, macromolecules are accumulated in the cytoplasm or periplasm^13,38^, while in ‘*Ca*. Uab amorphum’ the AcGFP1 signals of engulfed *E*. *coli* cells appear to be compartmentalized in PVs (Fig. 1j–o, Supplementary Movie 4).

Our microscopy studies show that ‘*Ca*. Uab amorphum’ can engulf various bacteria and picoeukaryotes, and appears to digest the engulfed organisms in specific compartments. The genomic analyses suggest that the organism may be able to utilize prey components such as sugar, amino acids and nucleotides from their prey. Therefore, we propose that engulfment by ‘*Ca*. Uab amorphum’ may serve a phagocytosis-like function.

Eukaryotic phagocytosis is regulated by many eukaryote-specific genes that are involved in recognition of prey, signal transduction, rearrangement of cytoskeleton, membrane remodelling and phagosome maturation^40^. ‘*Ca*. Uab amorphum’ possesses an actin-like gene that might be derived from an Asgard archaeon or a relative. However, we did not identify any other genes with homology to eukaryotic genes involved in phagocytosis. In addition, the structure of the ‘*Ca*. Uab amorphum’ PV seems to be different from that of the eukaryotic phagosome. Therefore, the phagocytosis-like process observed in ‘*Ca*. Uab amorphum’ is probably not homologous to eukaryotic phagocytosis, despite apparent morphological and functional similarities.

The endokaryotic hypothesis proposes that the origin of an ancestral eukaryote involved a Gram-negative bacterial host engulfing an archaeon, accounting for origin of the nucleus as an engulfed cell^41^. ‘*Ca*. Uab amorphum’ is unlikely to be a descendant of such Gram-negative bacterial host, but it may be a good bacterial model to test this hypothesis. Although it has been suggested that phagocytosis without mitochondria is implausible due to the energetic cost of phagocytic functions^42,43^, our study indicates that prokaryotes can take up other microorganisms in the absence of an energy-producing symbiont. Therefore, primitive phagocytosis may have originated in a mitochondrion-less eukaryote ancestor, with mitochondrion acquisition by phagocytosis being a later event.

Our analysis of environmental 16S rRNA gene data indicates that many uncultured lineages detected in various environments belong to the ‘*Ca*. Uab amorphum’ clade and have high genetic diversity. Therefore, it is tempting to speculate that some of these uncultured organisms may display other unusual phenotypes, may be acting as bacterial predators in marine, freshwater and terrestrial microbial ecosystems. Culture establishment and comparative analysis of Uab lineages will help to unveil their ecological importance and the evolution of eukaryotic-like features, and may provide insights about the origin of eukaryotes.

## Methods

### Data reporting

No statistical calculations were performed to pre-determine the sample size.

### Sample collection and culture establishment

Surface seawater was collected in the Republic of Palau (longitude = 7.181386° N, latitude = 134.336947° E) on 27 October 2015. Approximately 500 mL of the seawater sample was filtered through 5 µm Isopore membrane filters (Millipore, Billerica, USA), and was subsequently concentrated to approximately 10 mL using 0.6 µm Isopore membrane filters (Millipore, Billerica, USA). The concentrated sample was serially diluted across 12 wells by using a 96-well culture plate filled with ESM medium^44^. Samples were incubated at 24 °C in the dark for 10 days. ‘*Candidatus* Uab amorphum’ and other bacterial cells were observed in several wells under light microscope following the incubation period. Single ‘*Ca*. Uab amorphum’ cells were isolated from the incubated sample with a micropipette, and were transferred into a new 96-well cell culture plate filled with the ESM medium. A small amount of the incubated sample was also added in the new 96-well cell culture plate together with isolated ‘*Ca*. Uab amorphum’ cell in order to inoculate bacteria as food sources. As a result, a xenic culture that consists of clonal ‘*Ca*. Uab amorphum’ and unidentified bacteria was established. The xenic culture was maintained in a culture flask or a culture plate in ESM medium at 20 °C in the dark, and was subcultured into A new medium every month.

To establish a monoxenic culture of ‘*Ca*. Uab amorphum’, *Alteromonas macleodii* (NBRC 102226), which was obtained from NITE Biological Resource Centre (NBRC), was inoculated into a 96-well culture plate filled with the ESM medium. A single cell of ‘*Ca*. Uab amorphum’ isolated by the micropipette was added into the culture plate. The monoxenic culture of ‘*Ca*. Uab amorphum’ was maintained in the same condition as the xenic culture. No antibodies were used during the culture establishment. The monoxenic culture was deposited at the Japan Collection of Microorganisms (JCM) as JCM 39082.

### Light and time-lapse microscopic observation

Cells of ‘*Ca*. Uab amorphum’ and unidentified prey bacteria in the xenic culture were inoculated into glass-bottomed dishes filled with the ESM medium. Cells were incubated for 1–5 days prior to observation. Light micrographs and time-lapse videos were taken using an Olympus IX71 inverted microscope (Olympus, Tokyo, Japan) equipped with an Olympus DP73 CCD camera (Olympus, Tokyo, Japan). Sodium azide (76.9 mM) was added to the xenic culture 15 min before observation. For the feeding experiment, cells of *Debaryomyces hansenii* JCM 1439, *Lactobacillus farciminis* JCM 1097 and *Staphylococcus condimenti* JCM 6074 (obtained from JCM) were added to glass-bottomed dishes together with ‘*Ca*. Uab amorphum’ and observed immediately under light microscope. Cells of *Bathycoccus prasinos* NIES-2670 (obtained from the National Institute for Environmental Studies, NIES) were added to glass-bottomed dishes together with ‘*Ca*. Uab amorphum’, and were incubated for one day prior to light microscopic observation and specimen preparation for transmission electron microscopic (TEM) observation.

### DNA extraction, PCR and sequencing

Cells of ‘*Ca*. Uab amorphum’ and unidentified prey bacteria in the xenic culture were collected by centrifugation. Total DNA was extracted using the DNeasy plant mini kit (Qiagen, Hilden, Germany) according to the manufacturer’s instructions. PCR was performed using primer pairs specific for the bacterial 16S rRNA gene (27F and 1492R)^45,46^. The PCR cycles (30 cycles) consisted of denaturation at 96 °C for 10 s, annealing at 55 °C for 30 min and extension at 68 °C for 2 min. The amplicon was purified with the QIAquick Gel Extraction kit (Qiagen, Hilden, Germany), and was then cloned into the p-GEM T-easy vector (Promega, Tokyo, Japan). Ten clones were completely sequenced with a 3130 Genetic Analyser (Applied Biosystems, Foster City, CA), using the BigDye Terminator v3.1 cycle sequencing kit (Applied Biosystems). Sequenced clones were assembled into six sequences by the CodonCode Aligner (CodonCode Co., Centerville, MA) with a sequence similarity threshold of 99%. The 16-S rRNA gene sequence of ‘*Ca*. Uab amorphum’ was deposited in the GenBank database with accession code LC496071.

### Fluorescent microscopy

Fluorescein isothiocyanate (FITC)-labelled oligonucleotide probes EUB338 (ref. ^47^) and PLA886 (ref. ^48^) were purchased from FASMAC Co., Ltd. (Kanagawa, Japan). EUB338 completely matched all sequences derived from the xenic culture, while PLA886 matched only to the 16S rRNA gene sequence of ‘*Ca*. Uab amorphum’, and had at least three mismatches to other sequences (Supplementary Fig. 2) The xenic culture of ‘*Ca*. Uab amorphum’ was cultured on coverslips that were treated with 0.1% (w/v) poly L-lysine (Sigma Chemical Co., St. Louis, MO) for one week prior to observation. Fixation and hybridization were performed as described in a previous study^47^. Cells grown on coverslips were fixed with 4% (w/v) paraformaldehyde for 16 h at 4 °C, and were then treated with a series of 50%, 80% and 96% ethanol for 3 min each and air-dried. Hybridization buffer containing 0.9 M NaCl, 20 mM Tris/HCl (pH 8.0), 0.01% (w/v) SDS, 5 ng/μL probe and a specific amount of formamide (Supplementary Table 12) were mounted on coverslips and incubated at 46 °C for 1.5 h in humid chambers. In the case of PLA886, an equimolar amount of a competitor oligonucleotide (cPLA886) was added to the hybridization buffer to avoid binding to non-target bacteria. Coverslips were washed in pre-heated (48 °C) washing buffer containing 0.9 M NaCl, 20 mM Tris/HCl (pH 8.0) and 0.01% (w/v) SDS, and were then placed in the washing buffer for 20 min at 48 °C. They were then washed with distilled water and air-dried. Each coverslip was incubated with PBS containing 0.1 µg/mL 4′,6-diamidino-2-phenylindole (DAPI) for 10 min in the dark. Coverslips were washed with distilled water, air-dried, mounted with SlowFade Diamond (Invitrogen, Carlsbad, CA) and sealed with nail polish. Specimens were then observed using a Leica DMRD microscope (Leica, Wetzlar, Germany) equipped with an Olympus DP73 CCD camera (Olympus, Tokyo, Japan).

AcGFP1-labelled *Escherichia coli* was prepared by transformation using TOP10-competent cells (Thermo Fisher Scientific, MA, USA) and pAcGFP1 Vector (Takara, Tokyo, Japan). Cells of AcGFP1-labelled *E. coli* were added to glass-bottomed dishes together with ‘*Ca*. Uab amorphum’ and immediately observed under Nikon A1 confocal microscope (Nikon, Tokyo, Japan). For detection of acidic compartments in ‘*Ca*. Uab amorphum’ cells, ‘*Ca*. Uab amorphum’ and AcGFP1-labelled *E. coli* in glass-bottomed dishes were incubated with 1 μM L^−1^ LysoTracker Red DND-99 (Thermo Fisher Scientific) in the dark for 1 h. Cells were washed three times by ESM medium and observed under Nikon A1 confocal microscope. For detection of reactive oxygen species, ‘*Ca*. Uab amorphum’ in glass-bottomed dishes was incubated with 1 μM L^−1^ LysoTracker Red DND-99 and 1 mM L^−1^ DCFH-DA (OxiSelect Intracellular ROS Assay Kit, Cell Biolabs, CA, USA) in the dark for 1 h. Cells were washed three times by ESM medium and observed under Nikon A1 confocal microscope.

### Electron microscopy

For scanning electron microscopy, cells of ‘*Ca*. Uab amorphum’ were cultured on 8.5-mm-diameter glass SEM plates (Okenshoji Co., Tokyo, Japan) treated with 0.1% (w/v) poly L-lysine (Sigma Chemical Co., St. Louis, MO) for one week. Cells were pre-fixed with vapour of 4% (w/v) osmium tetroxide (OsO_4_) for 30 min, and were subsequently post-fixed with 1% (w/v) osmium tetroxide in culture medium for 2 h. Fixed cells were dehydrated in a series of 15–100% (v/v) ethanol. After dehydration, specimens were placed once in a 1:1 mixture of 100% ethanol and t-butyl alcohol, and then twice in 100% t-butyl alcohol, and chilled in the freezer. The specimens were freeze-dried using a VFD-21S (SHINKU-DEVICE, Ibaraki, Japan) freeze drier, and were then mounted on aluminium stubs using carbon paste. Specimens were sputter-coated with platinum–palladium using a Hitachi E-102 sputter-coating unit (Hitachi High-Technologies Corp., Tokyo, Japan), and were observed using a JSM-6360F field emission SEM (JEOL, Tokyo, Japan).

For TEM observation, cells were collected by centrifugation, and were pre-fixed with a mixture containing 1% glutaraldehyde, 0.1 M cacodylate buffer and 0.25 M sucrose for 1 h. Pelleted cells were washed twice with 0.2 M sodium cacodylate buffer (pH 7.2), and were post-fixed with a mixture containing 1% osmium tetroxide and 0.2 M cacodylate buffer. Fixed cells were dehydrated in a series of 30–100% (v/v) ethanol. After dehydration, cells were placed in a 1:1 mixture of 100% ethanol and acetone for 10 min, and 100% acetone for 10 min two times each. Resin replacement was performed with a 1:1 mixture of acetone and Agar Low Viscosity Resin R1078 (Agar Scientific Ltd., Stansted, England) for 30 min, and then with Low Viscosity Resin R1078 for 2 h. Resin was polymerized at 60 °C for 12 h. Ultra-thin sections were prepared on a Reichert Ultracut S ultramicrotome (Leica, Vienna, Austria). Cell sections were double-stained with 2% (w/v) uranyl acetate and lead citrate, and were observed using a Hitachi H-7650 electron microscope (Hitachi High-Technologies Corp., Tokyo, Japan) equipped with a Veleta TEM CCD camera (Olympus, Tokyo, Japan).

### Molecular phylogenetic analyses using 16S rRNA gene

We prepared a dataset containing 16S rRNA gene sequences of major bacterial lineages and ‘*Ca*. Uab amorphum’. The sequences were first automatically aligned in MAFFT v7.273^49^ with the G-INS-i algorithm at default settings, and were then manually edited with SeaView version 4.6^50^. Ambiguous regions in the alignment were trimmed with SeaView. The final alignment consisted of 71 OTUs and 1307 sites. The best substitution model was searched for using jModelTest 2.1.10^51^, and the GTR + Γ + I model was selected. A maximum likelihood (ML) tree was heuristically searched for using RAxML version 8.1.15^52^ under the GTR + Γ + I model. Tree searches began with 20 randomized maximum-parsimony trees, and the highest log likelihood (lnL) was selected as the ML tree. A nonparametric bootstrap analysis with 1000 replicates was conducted using RAxML under the GTR + Γ + I model. A Bayesian analysis was run using MrBayes 3.2.2^53^ with the GTR + Γ + I model for each dataset. One cold and three heated Markov chain Monte Carlo chains with default temperatures were run for 5 × 10^6^ generations; lnL values and trees were sampled at 100-generation intervals. The first 2.2 × 10^6^ generations with an average standard deviation of split frequencies (ASDSF) greater than 0.01 were discarded as “burnin”. Bayesian posterior probabilities (BPP) and branch lengths were calculated from the remaining trees.

In order to conduct molecular phylogenetic analysis of environmental sequences related to ‘*Ca*. Uab amorphum’, we prepared a 16 S rRNA gene dataset including major subgroups of Planctomycetes, which were selected based on a previous phylogenetic study^54^. We added several Chlamydiae and Verrucomicrobia OTUs to the dataset as outgroups. Environmental sequences related to ‘*Ca*. Uab amorphum’ were screened using the BLASTn program, with the 16S rRNA gene sequence of ‘*Ca*. Uab amorphum’ as query. The top 300 BLASTn hit sequences were added to the dataset. Sequences in the dataset were automatically aligned in MAFFT with the G-INS-i algorithm at default settings, and were then manually aligned and trimmed using SeaView. Preliminary maximum likelihood tree was constructed by RAxML with rapid bootstrap analysis (100 replicates) under a GTR + Γ model. We removed BLASTn-derived environmental sequences that did not form a clade with ‘*Ca*. Uab amorphum’ from the final dataset. Sequences in the final dataset were aligned and trimmed as shown above; the final alignment consisted of 100 OTUs and 1,351 sites. The best model was identified using jModelTest, and the GTR + Γ + I model was selected. The ML tree was heuristically searched for using RAxML under the GTR + Γ + I model. Tree searches began with 20 randomized maximum-parsimony trees, and the highest lnL was selected as the ML tree. A nonparametric bootstrap analysis with 1,000 replicates was conducted using RAxML under the GTR + Γ + I model. A Bayesian analysis was conducted using MrBayes with the GTR + Γ + I model for each dataset. One cold and three heated Markov chain Monte Carlo chains with default temperatures were run for 5 × 10^6^ generations; lnL values and trees were sampled at 100-generation intervals. The first 1.0 × 10^6^ generations with ASDSF greater than 0.01 were discarded as “burnin”. BPP and branch lengths were calculated from the remaining trees.

We screened for sequences related to ‘*Ca*. Uab amorphum’ from Tara Ocean 16S rRNA gene sequences^10^ using BLASTn, with the ‘*Ca*. Uab amorphum’ 16S rRNA gene sequence and environmental Uab clade sequences as query. We added sequences of BLASTn hits with sequence similarities above 97% and sequence length over 100 bp to the dataset used in the environmental 16S rRNA gene sequence analysis. Sequences in the dataset were automatically aligned in MAFFT with the G-INS-i algorithm at default settings, and were then manually aligned and trimmed using SeaView. Preliminary maximum likelihood tree was constructed by RAxML with rapid bootstrap analysis (100 replicates) under a GTR + Γ model. We removed environmental sequences that did not form a clade with ‘*Ca*. Uab amorphum’ from the final dataset. Sequences in the final dataset were aligned and trimmed as shown above; the final alignment consisted of 189 OTUs and 1,509 sites. The ML tree was heuristically searched for using RAxML under the GTR + Γ + I model. Tree searches began with 20 randomized maximum-parsimony trees, and the highest lnL was selected as the ML tree. A nonparametric bootstrap analysis with 1,000 replicates was conducted using RAxML under the GTR + Γ + I model.

### Genome sequencing

For genome sequencing, total DNA was extracted from the monoxenic culture of ‘*Ca*. Uab amorphum’ that was cultivated until most prey bacteria were consumed. Long reads (157,852 reads, 1.4 Gb) were sequenced using PacBio RS II (Pacific Biosciences, Melon Park, CA) with one cell (P6–C3 chemistry). Mean read length was 9.0 kbp. Sequencing was performed by Macrogen (Seoul, Korea).

### Genome assembly and annotation

Raw long reads were error-corrected and assembled using Canu v1.4, and 49 contigs were acquired. To remove prey sequences, homology search was performed using BLASTn against the NCBI nr database; only the longest contig was derived from *Ca*. Uab amorphum (Supplementary Table 2), which was manually circularized. Any other sequences, e.g. plasmids, were not found in ‘*Ca*. Uab amorphum’. The contig was polished using raw long reads by Pbjelly 0.3.0^55^ and Quiver 2.1.0 (Pacific Biosciences). Gene models were constructed using the DFAST web server^56^ with the Refseq database. Functional annotation was performed using the EggNOG mapper web server^57^.

### Molecular phylogenetic analysis using 171 proteins

Molecular phylogenetic analysis was performed using 171 highly conservative orthologues. The dataset was composed of 29 operation taxonomic units (OTU), which contained most available genomes from planctomycetes. Four species in Verrucomycrobia or Chlamydiae were used as an outgroup. The highly conserved orthologues were searched for by reciprocal blast best hit with the cut-off of coverage ≥30% and similarity ≥40%; 171 proteins (52,272 amino acids) were conserved among ≥90% of the OTU, and were used for the phylogenetic analysis. The amino acids were aligned using Mafft v7.221, and highly diversified regions were manually trimmed on MEGA 7^58^. Model test was performed using IQ-TREE 1.4.3^59^, following the Bayesian information criterion (BIC). Phylogenetic analysis was performed as described above using RAxML version 8.2.9. A nonparametric bootstrap analysis with 200 replicates under the LG + gamma + I model was performed. A Bayesian analysis was run using MrBayes 3.2.6 with the same model for 1.0 × 10^6^ generations; lnL values and trees were sampled at 1000-generation intervals. The initial 25% generations with ASDSF values greater than 0.01 were discarded as “burnin”. BPP and branch lengths were calculated from the remaining trees.

### Inference of digestive proteins in the FV

Subcellular localization of proteins was predicted using PSORTb 3.0^60^, Cello v.2.5^61^ and LocTree3^62^ with an organism option: Gram-negative bacteria or bacteria. Proteins were categorized into “secreted” or “extracellular” by at least one of the three prediction programmes that were considered a candidate FV protein. Digestive proteins were identified using the KEGG database. Digestive proteins for DNA/RNA were searched for using GO terms (GO:0004518). Digestive proteins for peptidoglycan were searched for using the HyPe web server^63^.

### Detection of horizontal gene transfer

Genes putatively derived by HGT were identified using HGTector v0.2.1^64^ with the following cut-off values: e-value = 1e–20, identity = 30 and coverage = 40. In this analysis, based on the phylogenetic relationships, “selfGroup” and “closeGroup” were set as ‘*Candidatus* Brocadiaceae’ (taxonomic ID: 1127830) and the PVC (Planctomycetes–Verrucomicrobia–Chlamydiae) group (taxonomic ID: 1783257), respectively.

In addition, we searched Uab homologues of the 347 eukaryote-specific proteins (ESPs)^21^ and actin-binding proteins^37^ in the *Saccharomyces* Genome Database^65^ by BLASTp with the cut-off: E-value < 1E–5. The Uab proteins with any hits were re-checked by BLASTp against the NCBI nr database.

### Molecular phylogenomic analyses of single proteins

For molecular phylogenetic analyses of single proteins (α-amylase, actin, acyloxyacyl hydrolase (AOAH), phospholipase C (PLC), diacylglycerol acyltransferase (DGAT), carboxypeptidase, DNase I and EPT1), we screened each protein sequence by BLASTp, against the NCBI nr database and constructed the dataset. Datasets were aligned by MAFFT v7.273, and were then manually edited with SeaView version 4.6 or MEGA 7. The final alignments consisted of 331 amino acid positions and 17 OTUs for α-amylase, 362 amino acid positions and 103 OTUs for actin, 534 amino acid positions and 28 OTUs for AOAH, 255 amino acid positions and 17 OTUs for PLC, 408 amino acid positions and 17 OTUs for DGAT, 206 amino acid positions and 6 OTUs for carboxypeptidase, 173 amino acid positions and 11 OTUs for DNase I and 154 amino acid positions and 10 OTUs for EPT1. ML trees were constructed using IQ-TREE 1.5.5 following the best-fit model, which was chosen in accordance with BIC (WAG + I + G4 for α-amylase, LG + G4 for actin, DGAT and DNase I, LG + I + G4 for AOAH and PLC, WAG + G4 for carboxypeptidase and mtZOA + I + G4 for EPT1) with 200 replicates of nonparametric bootstrap. A Bayesian analysis was run using MrBayes 3.2.6 with the LG + Γ model for the actin dataset. One cold and three heated Markov chain Monte Carlos with default temperatures were run for 1 × 10^7^ generations; lnL values and trees were sampled at 100-generation intervals. The first 7 × 10^6^ generations with an average standard deviation of split frequencies (ASDSF) value greater than 0.03 were discarded as “burnin”. Bayesian posterior probabilities (BPP) and branch lengths were calculated from the remaining trees.

### Description of ‘*Candidatus* Uab amorphum’

‘*Candidatus* Uab amorphum’ (U.a.b masc. n. a giant of Palauan mythology. a.mor’phus. L. neut. adj. amorphum amorphous, deformed).

Marine free-living aerobic Gram-negative bacterium was collected from surface seawater in the Republic of Palau (7.181386° N, 134.336947° E). Cells are flattened and round or oval shape with granular cytoplasm (Fig. 1a–c). Flagellum is absent. Cells were 3.2–7.8 μm (4.5 ± 0.85 μm) in the long axis and 2.8–5.5 μm (4.0 ± 0.57 μm) in the short axis (*n* = 79). When prey bacteria were abundant in the culture, cells occasionally reached 10 μm in diameter (Supplementary Fig. 1a). Cells attach to substrate and show gliding motility with changing shape (Supplementary Movie 1). Cells reproduce by binary fission. Periplasm (or paryphoplasm) highly invaginates into the cytoplasm (or pirellulosome) and shows a reticulate pattern (Fig. 3a–c). The cytoplasm includes multiple nuclear bodies (Fig. 3a, c). The cell interior includes four types of fibrous structures: large striated fibres, small striated fibres, short bundle fibres and cylindrical structures that contain linear fibres (Fig. 5). Cells can engulf bacteria and picoeukaryotes (*Bathycoccus prasinos*) (Fig. 1d–o, Fig. 3d–h, Supplementary Fig. 7, Supplementary Movies 2 and 4). Engulfed bacteria and algae can be found in phagosome-like vacuoles (PVs) (Fig. 2f–h, Supplementary Fig. 7s–t). Some PVs connect to other phagosome-like structures or outside of the cell by narrow ducts (Supplementary Fig. 8a, b). Similarity of the 16 S rRNA gene sequence (LC496071) to that of the closest species (the planctomycete ‘*Candidatus* Kuenenia stuttgartiensis’) is 79% (Supplementary Table 1). The genome of ‘*Ca*. Uab amorphum’ (AP019860) is circular and 9,503,110 bp in size. The G + C content of the genome is 39.4 mol%. A co-culture of ‘*Ca*. Uab amorphum’ has been deposited at the JCM as JCM 39082.

### Reporting summary

Further information on research design is available in the Nature Research Reporting Summary linked to this article.

## Supplementary information

Supplementary Information

Description of Additional Supplementary Files

Supplementary Data 1

Supplementary Data 2

Supplementary Movie 1

Supplementary Movie 2

Supplementary Movie 3

Supplementary Movie 4

Reporting Summary

## Data Availability

The 16S rRNA gene sequence of ‘*Ca*. Uab amorphum’ was deposited in DDBJ/GenBank/ENA with the accession number LC496071. Genome assembly was deposited in DDBJ/GenBank/ENA with accession number AP019860. Other relevant data are available in this article and its Supplementary Information files, or from the corresponding author upon request.
