# Supplementary material for: Phagocytosis-like cell engulfment by a planctomycete bacterium
Source: Nat Commun. 2019 Dec 11;10:5529. doi: 10.1038/s41467-019-13499-2 (PMC6906331; doi:10.1038/s41467-019-13499-2)
Supplement: Supplementary file 1 — Supplementary Information [file 41467_2019_13499_MOESM1_ESM.pdf]

Supplementary Information

Phagocytosis-like cell engulfment by a planctomycete bacterium

Shiratori et al.

Supplementary Table 1 | 16S rRNA gene sequences obtained from xenic culture of ó*Candidatus* Uab amorphum’

query	sequence length	blastn top hit*	identity
Contig 1 (Accession No.)<br>(= *Ca.* Uab amorphum)	1479 bp	ó*Candidatus* Kuenenia stuttgartiensis CT573071.1ô	79%
Contig 2 (= prey bacterium)	1455 bp	*Alteromonas* sp. strain SS12.31 KC160922.1	95%
Contig 3 (= prey bacterium)	1455 bp	*Alteromonas* sp. strain SS12.31 KC160922.1	99%
Contig 4 (= prey bacterium)	1455 bp	*Alteromonas* sp. strain CF6-3 FJ169998.1	99%
Contig 5 (= prey bacterium)	1463 bp	*Alcanivorax* sp. strain NT N57 AB166992.1	99%
Contig 6 (= prey bacterium)	1461 bp	*Pseudomonas pachastrellae* strain PTG4-14 EU603457.1	99%

* Uncultured/environmental sequences are excluded.

Supplementary Table 2 | Contigs derived from monoxetic culture of ‘*Candidatus* Uab amorphum’

contig name	length (bp)	number of reads	circular	sequence similarity with *Alteromonas macleodii* genome (%)	sequence similarity with ô*Ca.* Uab amorphumô16S rRNA gene (%)	remarks
tig00000001	9512535	37028	Yes*	75.49	100	Genome of ô*Ca.* Uab morphumô
tig00000060	261064	167	No	99.86	-	Genome of *Alteromonas macleodii*
tig00000062	234149	155	No	99.85	-	Genome of *A. macleodii*
tig00000065	273226	177	No	99.87	-	Genome of *A. macleodii*
tig00000067	226284	124	No	99.83	-	Genome of *A. macleodii*
tig00000069	21259	33	No	-	-	Possible contaminated sequence
tig00000070	160080	96	No	99.82	-	Genome of *A. macleodii*
tig00000072	134043	80	No	99.85	76.7	Genome of *A. macleodii*
tig00000073	125702	81	No	99.91	76.8	Genome of *A. macleodii*
tig00000076	122497	75	No	99.89	-	Genome of *A. macleodii*
tig00000078	106860	65	No	99.88	-	Genome of *A. macleodii*
tig00000080	118729	71	No	99.9	76.8	Genome of *A. macleodii*
tig00000082	131388	84	No	99.82	-	Genome of *A. macleodii*
tig00000084	93001	51	No	99.76	-	Genome of *A. macleodii*
tig00000087	49583	110	No	-	-	Possible contaminated sequence
tig00000088	94658	57	No	99.8	-	Genome of *A. macleodii*
tig00000091	104558	61	No	99.8	-	Genome of *A. macleodii*
tig00000093	98049	51	No	99.88	-	Genome of *A. macleodii*
tig00000095	75936	42	No	99.84	-	Genome of *A. macleodii*
tig00000098	89759	58	No	99.79	-	Genome of *A. macleodii*
tig00000101	82362	38	No	99.67	-	Genome of *A. macleodii*
tig00000103	71695	37	No	99.83	-	Genome of *A. macleodii*
tig00000104	77492	41	No	99.92	-	Genome of *A. macleodii*
tig00000107	77070	39	No	99.69	-	Genome of *A. macleodii*
tig00000109	64220	32	No	99.73	-	Genome of *A. macleodii*
tig00000111	68023	27	No	99.72	-	Genome of *A. macleodii*
tig00000112	52636	31	No	99.87	-	Genome of *A. macleodii*
tig00000113	61047	28	No	99.71	-	Genome of *A. macleodii*
tig00000118	57389	24	No	99.69	-	Genome of *A. macleodii*
tig00000120	40726	26	No	99.82	-	Genome of *A. macleodii*
tig00000121	50932	21	No	99.76	-	Genome of *A. macleodii*
tig00000123	62357	33	No	99.69	-	Genome of *A. macleodii*
tig00000126	38526	21	No	99.76	-	Genome of *A. macleodii*
tig00000127	47338	17	No	99.78	76.9	Genome of *A. macleodii*
tig00000128	43453	25	No	99.77	-	Genome of *A. macleodii*
tig00000132	63223	29	No	99.67	-	Genome of *A. macleodii*
tig00000134	52946	21	No	99.71	-	Genome of *A. macleodii*
tig00000135	56857	24	No	99.59	-	Genome of *A. macleodii*
tig00000136	31868	16	No	99.71	-	Genome of *A. macleodii*
tig00000140	48298	27	No	99.8	-	Genome of *A. macleodii*
tig00000143	42676	23	No	99.4	76.8	Genome of *A. macleodii*
tig00000145	40438	18	No	99.74	-	Genome of *A. macleodii*
tig00000147	30108	13	No	99.52	-	Genome of *A. macleodii*
tig00000148	32818	24	No	99.58	-	Genome of *A. macleodii*
tig00000151	27296	7	No	99.64	-	Genome of *A. macleodii*
tig00000152	26359	12	No	99.78	-	Genome of *A. macleodii*
tig00000153	36697	15	No	99.77	-	Genome of *A. macleodii*
tig00000175	24132	10	No	99.66	-	Genome of *A. macleodii*
tig00000178	22849	3	No	98.99	-	Genome of *A. macleodii*

*Circulized length; 9,503,110 bp

Supplementary Table 3 | Genome characteristics of ‘*Candidatus* Uab amorphum’ and eight planctomycete bacteria

	ó*Ca.* Uab amorphumô	*I. pallida*	*S. acidiphila*	*P. limnophila*	*P. staleyi*	*R. baltica*	*Gemmata* sp.	*R. brasiliensis*	*P. mikurensis*
strain	SRT547	ATCC43644	DSM 18658	DSM 3776	DSM 6068	SH1	SH-PL17	DSM 5305	NBRC 102666
Genome size (Mbp)	9.5	5.5	9.8	5.4	6.2	7.2	9.1	6	3.9
Number of plasmids	0	1	3	1	0	0	0	0	1
GC%	39.4	62.4	62.3	53.7	57.5	55.4	64.2	56.4	73.2
Number of CDSs	6,660	3888	7369	4301	4596	7325	7312	4719	3060
Number of rRNAs	6	9	24	4	3	3	9	6	3
Number of tRNAs	75	47	64	59	44	76	70	45	46
Average Protein Length (aa)	422.5	400.7	362.8	358.9	382.4	315.7	344.7	362.2	371.7
Coding Ratio (CDSs)	91.5	85.0	81.8	85.8	85.0	96.3	83.1	85.5	87.5
Reference seqences		NC_014962.1<br>NC_014957.1	NC_019892.1<br>NC_019893.1<br>NC_019894.1<br>NC_019895.1	NC_014148.1<br>NC_014149.1	NC_013720.1	NC_005027.1	NZ_CP011271.1	NC_015174.1	NC_017080.1<br>NC_017081.1

Supplementary Table 4 | Genes related to protein transport

gene symbol	protein ID					
**Type II secretion system**							
GspD	UABAM_02558	UABAM_04083	UABAM_06223	UABAM_06224	UABAM_06602		
GspF	UABAM_00096						
GspG	UABAM_00092	UABAM_00095	UABAM_01505	UABAM_03303	UABAM_04783		
GspJ	UABAM_06062						
GspK	UABAM_01912						
GspE	UABAM_01005	UABAM_01007	MPLA_01236	UABAM_01321	UABAM_01463	UABAM_06138	UABAM_06222
GspO	UABAM_01995						
**Sec-SRP**							
SecD/F	UABAM_02674	UABAM_04093					
SecY	UABAM_03972						
YidC	UABAM_00120						
SecA	UABAM_01766	UABAM_03633	UABAM_03639				
FtsY	UABAM_01922						
ffh	UABAM_06162						
**Twin arginine targeting (Tat)**							
TatC	UABAM_01580						

Supplementary Table 5 | Genes related to Peptidoglycan biosynthesis

	Gene symbol	protein ID	
		blastp hit of "*Ca*. Uab amorphum" proteins (e-value)	blastp query (*Escherichia coli* K-12 MG1655)
	MurA	UABAM_03634 (6.12e-86), UABAM_03143 (3.72e-4)	NP_417656
	MurB	UABAM_01805 (6.16e-14)	NP_418403
	MurC	UABAM_01806 (6.03e-79)	NP_414633
	MurD	UABAM_00997 (3.95e-38), UABAM_01806 (9.01e-10)	NP_414630
	MurE	UABAM_01806 (1.69e-11), UABAM_00997 (1.19e-7)	NP_414627
	MurF	UABAM_01806 (5.59e-10), UABAM_00997 (2.81e-8)	NP_414628
	MraY	UABAM_01808 (2.39e-57), UABAM_06107 (9.87e-3)	NP_414629
	MurG	UABAM_01807 (1.34e-32)	NP_414632
	MurJ/MviN	UABAM_01276 (5.26e-42), UABAM_00844 (2.2e-4)	NP_415587
	MtgA		NP_417675
low-molecular-weight PBP	dacA		NP_415165
	dacB	UABAM_04933 (1.42e-48)	NP_417649
	dacC		NP_415360
	dacD	UABAM_04185 (9.92e-2)	NP_416514
	yfew	UABAM_03436 (3.27e-18), UABAM_05443 (1.54e-16), UABAM_00445 (5.77e-15)	NP_416925
classA PBP	mrcA		NP_417855
	mrcB		NP_414691
	pbpC		NP_417014
classB PBP	mrdA		NP_415168
	ftsl		NP_414626

Supplementary Table 6 | Number of HGT-derived genes and thrir possible donors*

	number of genes
HGT-derived genes	
Archaea	43
Crenarchaeota	1
Euryarchaeota	40
Thaumarchaeota	2
Eukarya	2
Ascomycota	2
Bacteria	1778
Acidobacteria	31
Actinobacteria	40
Aquificae	8
Armatimonadetes	2
Bacteroidetes	239
Balneolaeota	14
Calditrichaeota	17
Candidatus Kryptonia	19
Chlorobi	8
Chloroflexi	46
Chrysiogenetes	1
Cyanobacteria	172
Deferribacteres	10
Deinococcus-Thermus	11
Elusimicrobia	3
Fibrobacteres	6
Firmicutes	326
Fusobacteria	3
Gemmatimonadetes	2
Ignavibacteriae	25
Nitrospinae	9
Nitrospirae	16
Proteobacteria	689
Spirochaetes	39
Synergistetes	8
Thermodesulfobacteria	9
Thermotogae	10
Total	1823
non HGT-derived genes	4777
Total protein coding genes	6600

* search cutoffs: E-value < 1E-20, identity > 30%, and coverage > 40%

Supplementary Table 7 | Eukaryote signature proteins in ô*Candidatus* Uab amorphumô genome.

			blastp querying *S. cerevisie* proteins against ô*Ca*. Uab amorphumôproteins		blastp querying ô*Ca*. Uab amorphumôproteins against NCBI nr database	
	Gene symbol	*S. cerevisie* protein ID	blast top hit (ô*Ca*. Uab amorphumôproteins)	e-value	blast top hit (nr) (removing hypothetical proteins and data from metagenome)	e-value
Actin	Act1	YFL039C	UABAM_01738	1.15E-75	actin [Cordyceps confragosa RCEF 1005]	7.00E-75
Actin-related	Arp1	YHR129C	UABAM_01738	2.54E-39	actin [Cordyceps confragosa RCEF 1005]	7.00E-75
	Arp2	YDL029W	UABAM_01738	4.99E-45	actin [Cordyceps confragosa RCEF 1005]	7.00E-75
	Arp3	YJR065C	UABAM_01738	9.09E-29	actin [Cordyceps confragosa RCEF 1005]	7.00E-75
	Arp4	YJL081C	UABAM_01738	6.06E-08	actin [Cordyceps confragosa RCEF 1005]	7.00E-75
	Arp5	YNL059C	UABAM_01738	0.000000502	actin [Cordyceps confragosa RCEF 1005]	7.00E-75
	Arp6	YLR085C	UABAM_01738	0.00000021	actin [Cordyceps confragosa RCEF 1005]	7.00E-75
Translation factors	Cam1	YPL048W	UABAM_05207	0.000000073	glutathione S-transferase family protein [Lentisphaera araneosa]	2.00E-52
GTP-binding proteins	Rho2	YNL090W	UABAM_04157	0.000000774	GTPase [Fibrobacter succinogenes]	2.00E-78
	Ypt6	YLR262C	UABAM_02845	0.000000246	GTP-binding protein [Mastigocoleus testarum]	0
	Arl3	YPL051W	UABAM_06593	0.000000489	WD40 repeat [Candidatus Kryptonium thompsoni]	4.00E-47
Cyclin	Cak1	YFL029C	UABAM_06662	3.22E-12	Stk1 family PASTA domain-containing Ser/Thr kinase [Firmicutes bacterium AF12-30]	6.00E-41
Kinases and phosphatases	Cdc7	YDL017W	UABAM_00443	4.35E-12	tetratricopeptide repeat protein [Sphaerospermopsis reniformis]	5.00E-116
	Sky1	YMR216C	UABAM_05439	2.21E-09	serine/threonine protein kinase [Patulibacter americanus]	2.00E-37
	Iks1	YJL057C	UABAM_01928	4.21E-15	tetratricopeptide repeat protein [Candidatus Aminicenantes bacterium]	2.00E-49
	Ykl171w	YKL171W	UABAM_03534	2.01E-09	serine/threonine protein kinase [Rhodopirellula sp.]	6.00E-62
	Vps15	YBR097W	UABAM_03878	0.00000015	serine/threonine protein kinase [Rubinisphaera sp.]	2.00E-52
	Kns1	YLL019C	UABAM_00315	5.22E-15	serine/threonine protein kinase [Nonomuraea sp. KC333]	6.00E-41
	Cdc14	YFR028C	UABAM_01136	1.33E-12	protein tyrosine phosphatase [Candiadtus Scalindua japonica]	2.00E-40
	Cdc55	YGL190C	UABAM_04730	0.000000328	NACHT nucleoside triphosphatase [Penicillium camemberti]	0
	Sds22	YKL193C	UABAM_03630	1.36E-13	leucine rich repeat protein [Leptospira noguchii]	3.00E-67
Histone-associated	Hat2	YEL056W	UABAM_04296	1.14E-15	(Myosin heavy-chain) kinase., Histone acetyltransferase [Haliscomenobacter hydrossis DSM 1100]	0
Nuclear pore and transport	Glc2	YDR477W	UABAM_04480	1.74E-32	Serine/threonine protein kinase [Planctomicrobium piriforme]	7.00E-76
	Srp1	YNL189W	UABAM_02812	0.000000279	TIR domain-containing protein [Microcystis aeruginosa]	2.00E-143
Clusters of unknown proteins	Msi1	YBR195C	UABAM_01649	4.78E-11	nacht and wd40 domain protein, partial [Umbilicaria pustulata]	0
	Rsa2	YMR131C	UABAM_04730	1.62E-08	NACHT nucleoside triphosphatase [Penicillium camemberti]	0
	Ykl121w	YKL121W	UABAM_01336	2.02E-11	putative WD repeat-containing protein, partial [Alternaria tenuissima]	2.00E-169
	Ymr102c	YMR102C	UABAM_05614	4.77E-11	putative WD repeat-containing protein, partial [Alternaria tenuissima]	1.00E-90
	Yor289w	YOR289W	UABAM_03205	1.13E-14	AmmeMemoRadiSam system protein A [delta proteobacterium PSCGC 5296]	3.00E-45
	Yer082c	YER082C	UABAM_04296	1.47E-10	(Myosin heavy-chain) kinase., Histone acetyltransferase [Haliscomenobacter hydrossis DSM 1100]	0
	Ypl247c	YPL247C	UABAM_02051	0.00000013	tetratricopeptide repeat protein [Ktedonobacter racemifer]	0
	Ypl236c	YPL236C	UABAM_06120	6.39E-12	Serine/threonine-protein kinase PknB [Planctomyces sp. SH-PL14]	8.00E-91
	Ylr409c	YLR409C	UABAM_04996	1.86E-19	(Myosin heavy-chain) kinase., Histone acetyltransferase [Haliscomenobacter hydrossis DSM 1100]	0
	Ymr068w	YMR068W	UABAM_02159	0.000000371	ankyrin repeat domain-containing protein [Fischerella sp. PCC 9605]	7.00E-49
	Ptk1	YKL198C	UABAM_02156	1.92E-18	serine/threonine protein kinase [Candidatus Solibacter usitatus Ellin6076]	4.00E-50

Eukaryotic sequence are highlighted in yellow.

Supplementary table 8 | Results of the feeding experiments of óCa. Uab amorphumô

Taxonomic group	Species	Strain	Gram staining	feeding
Bacteria, Proteobacteria	*Alteromonas macleodii*	NBRC 102226	negative	+
Bacteria, Proteobacteria	*Escherichia coli*	TOP10	negative	+
Bacteria, Firmicutes	*Lactobacillus farciminis*	JCM 1097	positive	+
Bacteria, Firmicutes	*Staphylococcus condimenti*	JCM 6074	positive	+
Eukarya, Chlorophyta	*Bathycoccus prasinos*	NIES-2670	n/a	+
Eukarya, Fungi	*Debaryomyces hansenii*	JCM 1439	n/a	-

Supplementary Table 9 | Proteins of ó*Ca.* Uab amorphumô that are putatively involved with digestion.

	protein ID	gene name	EC number	putative protein localization*
Sugar (glycogen) degradation	UABAM_05595	α-amylase	3.2.1.1	Extracellular
	UABAM_05988	α-amylase	3.2.1.1	Extracellular
Protein digestion	UABAM_06464	carboxypeptidase	3.4.17.15	Outer membrane
	UABAM_04121	S9 familiy peptidase	3.4.14.5	Extracellular
Phospholipid digestion	UABAM_00798	Phospholipase C	3.1.4.3	Extracellular
DNA/RNA digestion	UABAM_04815	Deoxyribonuclease I	3.1.21.1	Extracellular
	UABAM_00289	5'-nucleotidase	3.1.3.5	Outer membrane
Peptidoglycan digestion	UABAM_06081	N-acetylmuramoyl-L-alanine amidase	3.5.1.28	Extracellular

*predicted by Psortb, Cello, and LocTree3

Supplementary Table 10 | Proteins putatively related to antibiotic resistance

protein ID	eggNOG annotation	HGT (HGTector)*
UABAM_01413	multidrug transporter AcrB	1929465 (Idiomarina sp. 5.13)
UABAM_01641	multidrug transporter AcrB	765910 (Marichromatium purpuratum 984)
UABAM_02753	multidrug transporter AcrB	
UABAM_03431	multidrug transporter AcrB	1544451 (Thalassospira sp. TSL5-1)
UABAM_05788	multidrug transporter AcrB	
UABAM_06233	multidrug transporter AcrB	207559 (Desulfovibrio alaskensis G20)
UABAM_00787	AcrB AcrD AcrF family protein	1305731 (Marinobacter excellens HL-55)
UABAM_05187	AcrB/AcrD/AcrF family	880073 (Caldithrix abyssi DSM 13497)
UABAM_02471	AcrB/AcrD/AcrF family	880073 (Caldithrix abyssi DSM 13497)
UABAM_00787	acriflavin resistance protein	1305731 (Marinobacter excellens HL-55)
UABAM_01648	acriflavin resistance protein	
UABAM_03870	acriflavin resistance protein	
UABAM_04012	acriflavin resistance protein	
UABAM_04776	acriflavin resistance protein	1121391 (Desulfacinum infernum DSM 9756)
UABAM_06283	acriflavin resistance protein	
UABAM_02128	acriflavine resistance protein B	1266370 (Nitrospina gracilis 3/211)
UABAM_03163	aminoglycoside phosphotransferase	1821260 (Bacillus sp. NC2-31)
UABAM_04125	aminoglycoside phosphotransferase	
UABAM_06663	aminoglycoside phosphotransferase	
UABAM_00313	beta-lactamase	
UABAM_00445	beta-lactamase	
UABAM_00904	beta-lactamase	1664068 (bacterium 336/3)
UABAM_01671	beta-lactamase	
UABAM_03436	beta-lactamase	
UABAM_03539	beta-lactamase	309807 (Salinibacter ruber DSM 13855)
UABAM_04228	beta-lactamase	1302690 (Cnuella takakiae)
UABAM_04234	beta-lactamase	1173024 (Fischerella sp. PCC 9605)
UABAM_05381	beta-lactamase	1123277 (Spirosoma panaciterrae DSM 21099)
UABAM_05443	beta-lactamase	402881 (Parvibaculum lavamentivorans DS-1)
UABAM_06005	beta-lactamase	1997 (Microtetraspora fusca)
UABAM_00326	beta-lactamase domain protein	1246637 (Desulfamplus magnetovallimortis)
UABAM_00939	beta-lactamase domain protein	1235591 (Pseudorhodoplanes sinuspersici)
UABAM_01747	beta-lactamase domain protein	1286635 (Desulfotignum phosphitoxidans DSM 13687)
UABAM_01963	beta-lactamase domain protein	1633631 (Candidatus Kryptonium thompsoni)
UABAM_06106	beta-lactamase domain-containing protein	1150474 (Mesoaciditoga lauensis cd-1655R = DSM 25116)
UABAM_00685	lactamase_B	927787 (Microbulbifer mangrovi)
UABAM_00892	metallo-beta-lactamase	675511 (Methylomicrobium buryatense 5G)
UABAM_05568	metallo-beta-lactamase	1201290 (Bacteriovorax sp. BAL6_X)
UABAM_00823	metallo-beta-lactamase superfamily	
UABAM_03770	metallo-beta-lactamase superfamily	1664068 (bacterium 336/3)
UABAM_05072	metallo-beta-lactamase superfamily	1958811 (Leptospira sp. CLM-U50)
UABAM_05384	metallo-beta-lactamase superfamily	1184267 (Bdellovibrio exovorus JSS)
UABAM_06572	metallo-beta-lactamase superfamily	
UABAM_00820	cation multidrug efflux pump	
UABAM_02165	chloramphenicol acetyltransferase	318464 (Clostridium sulfidigenes)
UABAM_00466	chloramphenicol resistance protein	1498499 (Legionella norrlandica)
UABAM_05970	MATE efflux family protein	1122189 (Malonomonas rubra DSM 5091)
UABAM_05659	MATE efflux family protein	926566 (Terriglobus roseus DSM 18391)
UABAM_03864	MATE efflux family protein	262489 (delta proteobacterium MLMS-1)
UABAM_00859	MATE efflux family protein	1192034 (Chondromyces apiculatus DSM 436)
UABAM_01667	multidrug resistance protein	
UABAM_05343	multidrug resistance protein	
UABAM_04245	multidrug resistance protein msba	
UABAM_05659	putative multidrug resistance protein NorM	
UABAM_01784	tetracycline efflux Na+/H+ antiporter family transporter Tet(35)	1121429 (Desulfotomaculum putei DSM 12395)
UABAM_06437	tetracycline resistance MFS efflux pump	1849360 (Balneola sp. EhC07)

* NCBI taxon ID and name of related taxa are shown.

Supplementary Table 11 | Actin binding proteins in óCa. Uab amorphumô genome

		blastp querying S. cerevisie proteins against óCa . Uab amorphumôproteins		blastp querying óCa . Uab amorphumôproteins against NCBI nr database	
Gene symbol	*S. cerevisie* protein ID	blast top hit (óCa . Uab amorphumôproteins)	e-value	blast top hit (nr) (removing hypothetical proteins and data from metagenome)	e-value
Abp1	YCR088W	(no hit)			
Abp140	YOR239W	(no hit)			
Aip1	YMR092C	UABAM_01722	9.71E-12	TIR domain-containing protein [Sorangium cellulosum]	1.00E-84
Bni1	YNL271C	(no hit)			
Bnr1	YIL159W	(no hit)			
Cap1	YKL007W	(no hit)			
Cap2	YIL034C	(no hit)			
Crn1	YLR429W	UABAM_04996	1.50E-18	(Myosin heavy-chain) kinase., Histone acetyltransferase [Haliscomenobacter hydrossis DSM 1100]	0
Iqg1/Cyk1	YPL242C	(no hit)			
Myo5	YMR109W	(no hit)			
Myo1	YHR023W	(no hit)			
Myo2	YOR326W	(no hit)			
Myo4	YAL029C	(no hit)			
Pan1	YIR006C	(no hit)			
Sac6	YDR129C	(no hit)			
Scp1	YOR367W	(no hit)			
Sla2	YNL243W	(no hit)			
Tpm1	YNL079C	(no hit)			
Tpm2	YIL138C	(no hit)			
Bud6/Aip3	YLR319C	(no hit)			
Las17/Bee1	YOR181W	(no hit)			
Pfy1	YOR122C	(no hit)			
Srv2	YNL138W	(no hit)			
Cof1	YLL050C	(no hit)			
Twf1	YGR080W	(no hit)			
Vrp1	YLR337C	(no hit)			

Supplementary Table 12 | Oligonucleotide probes used in the fluorescent in situ hybridization

probe name	sequence (5'-3')	binding position in *Escherichia coli* 16S	amount of formamide*	reference
EUB338	GCT GCC TCC CGT AGG AGT	338–355	0	1
PLA886†	GCC TTG CGA CCA TAC TCC C	886–904	35%	2

* Formamide concentrations optimised in references are used in this study.

† Hybridised with equimolar amount of competitor oligonucleotide cPLA886 (5' GCC TTG CGA CCG TAC TCC C 3') to avoid binding to nontarget bacteria.

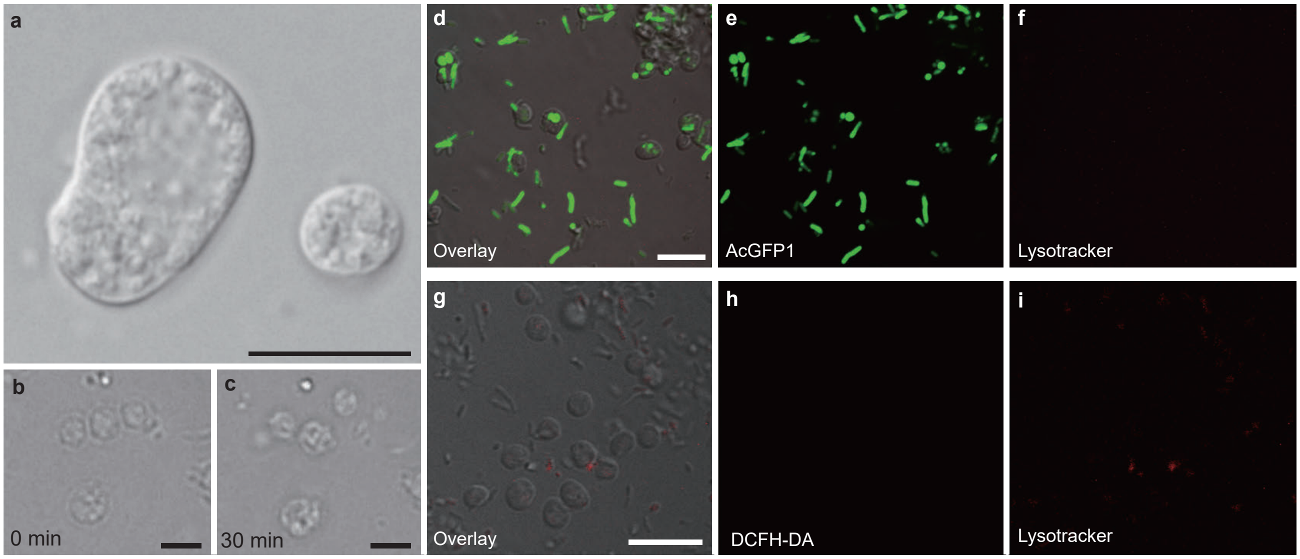

Supplementary Figure 1 | Light and fluorescent micrographs of ó*Candidatus* Uab amorphumô. **(a)** Light micrograph of large cell of óCa. Uab amorphumô. **(b,c)** Selected images of time-lapse video micrographs showing immotile cells under sodium azide. **(d–f)** Confocal fluorescent micrographs showing that low pH (red) signal is not observed in cells and does not overlap with AcGFP1 signal of engulfed *Escherichia coli*. **(g–i)** Confocal fluorescent micrographs showing there is no low pH signal (red) and reactive oxygen species (green) signal in cells. Scale bars, 10 µm **(a,d–i)** and 5 µm **(b,c)**.

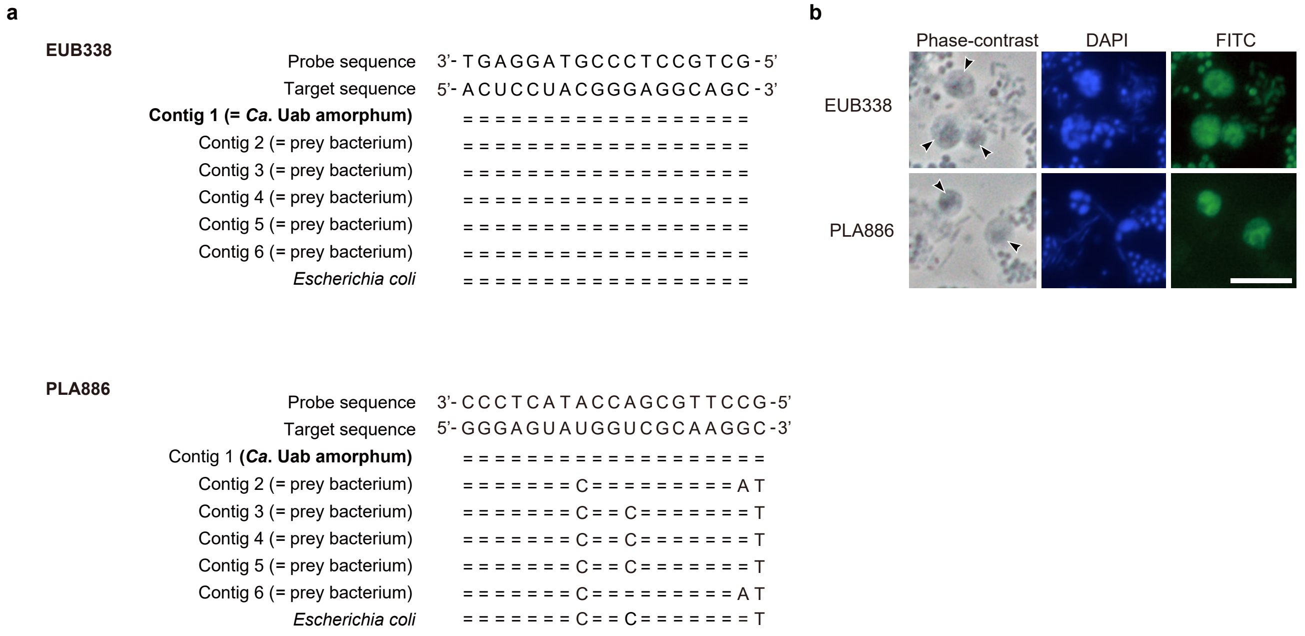

Supplementary Figure 2 | Fluorescence in situ hybridization experiment on ó*Candidatus* Uab amorphumô and prey bacteria cells in co-culture. (**a**) Alignments of oligonucleotide probes and 16S rRNA gene sequences derived from a xenic culture of ó*Ca*. Uab amorphumô. (b) Fluorescent micrographs of the xenic culture of ó*Ca*. Uab amorphumô using oligonucleotide probes EUB338 (upper) and PLA886 (lower). EUB338 stained all bacterial cells in the culture. PLA886 stained cells of ó*Ca*. Uab amorphum. Arrowheads indicate cells of ó*Ca*. Uab amorphumô.

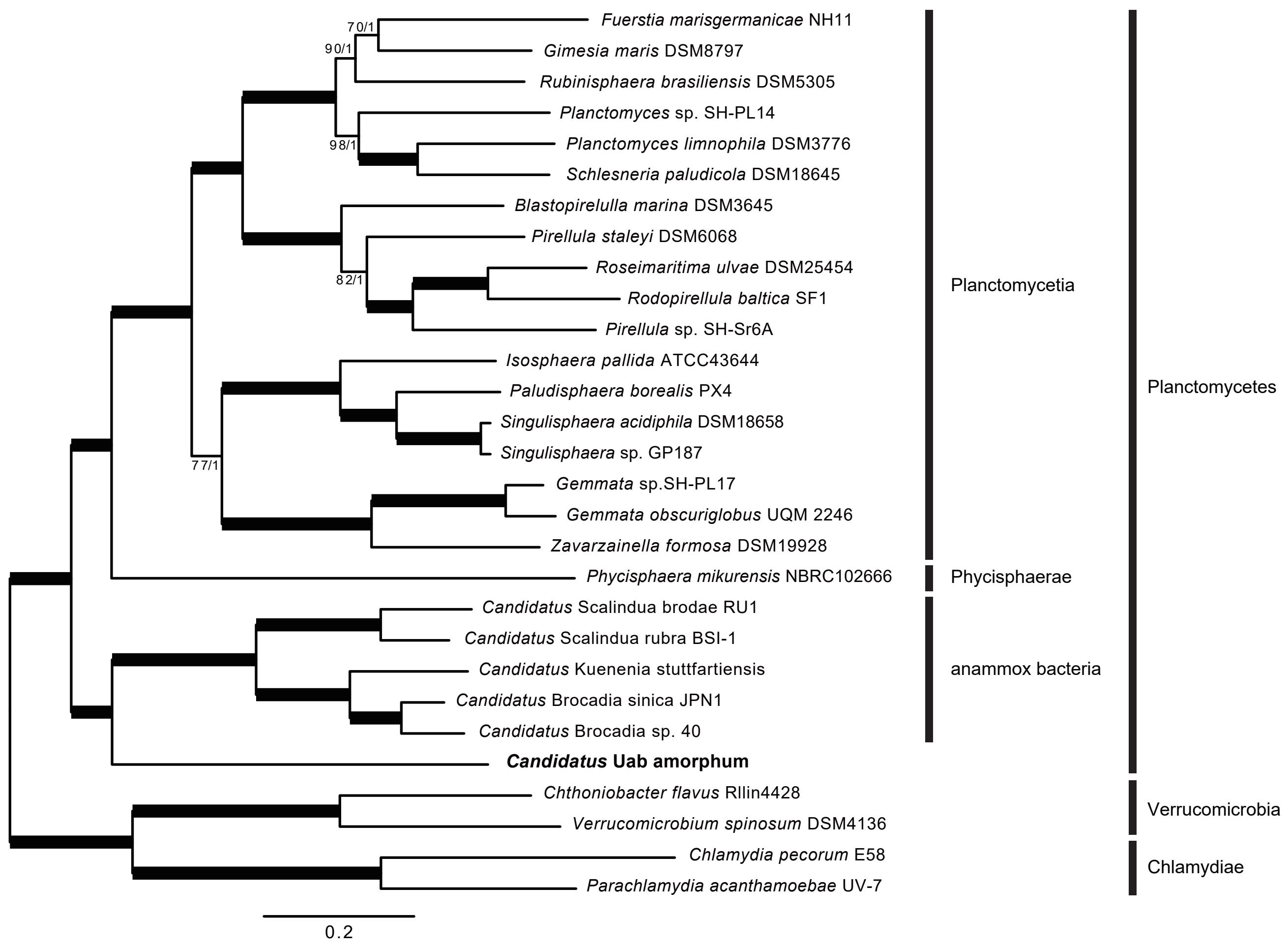

Supplementary Figure 3 | Maximum likelihood tree of 171 proteins. The tree was inferred from 52,272 amino acid positions of 29 taxa. Left and right values on nodes indicate bootstrap value and Bayesian posterior probability, respectively. Bold branches indicate maximum supports of both bootstrap value and Bayesian posterior probability. Scale bar indicates nucleotide substitution rate per site.

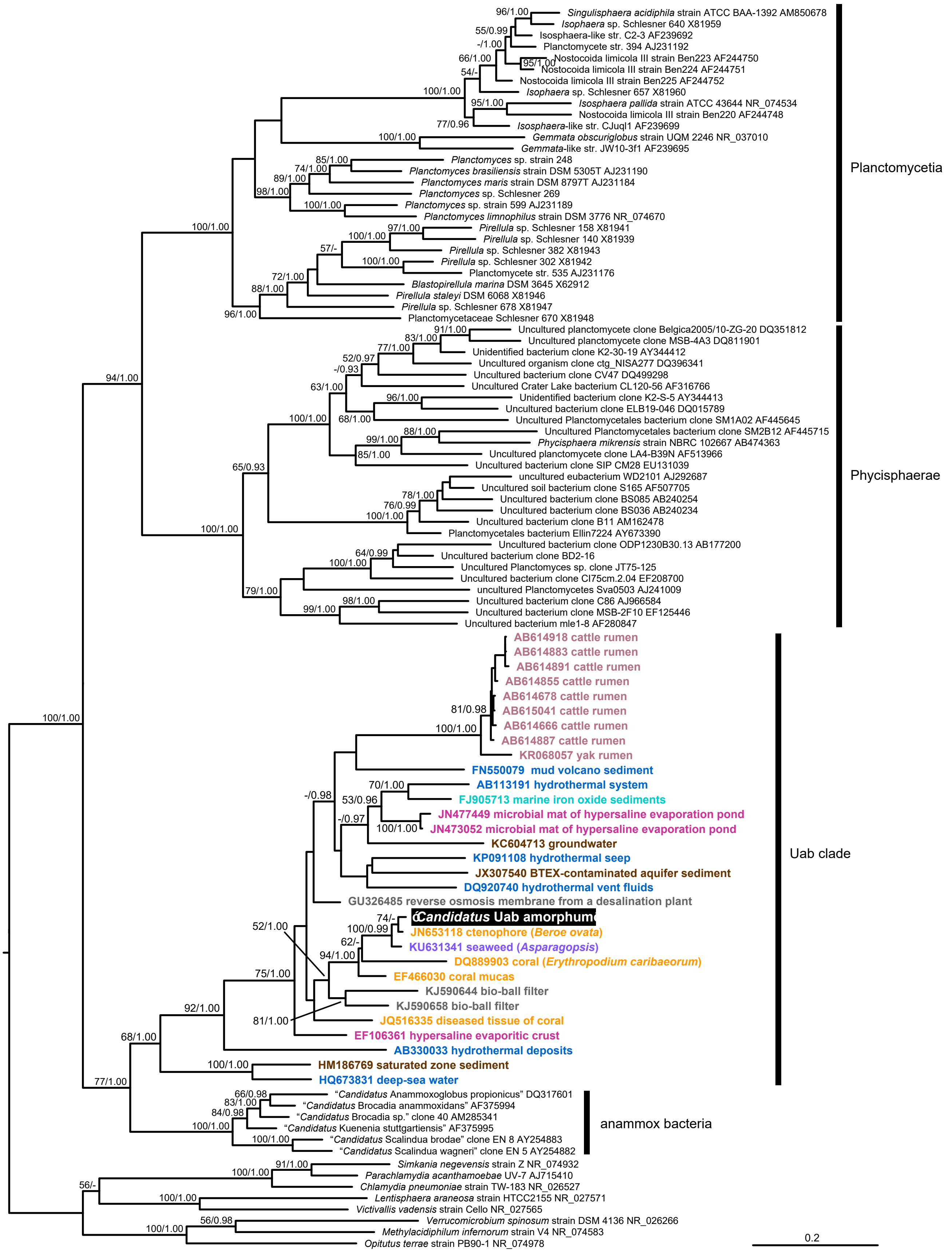

Supplementary Figure 4 | Maximum likelihood tree of 16S rRNA gene sequences including environmental sequences close to ó*Candidatus* Uab amorphumô. The tree was inferred from 1,351 nucl eotide positions of 100 taxa. Left and right values on nodes indicate bootstrap value and Bayesian posterior probability, respectively. Scale bar indicates nucleotide substitution rate per site.

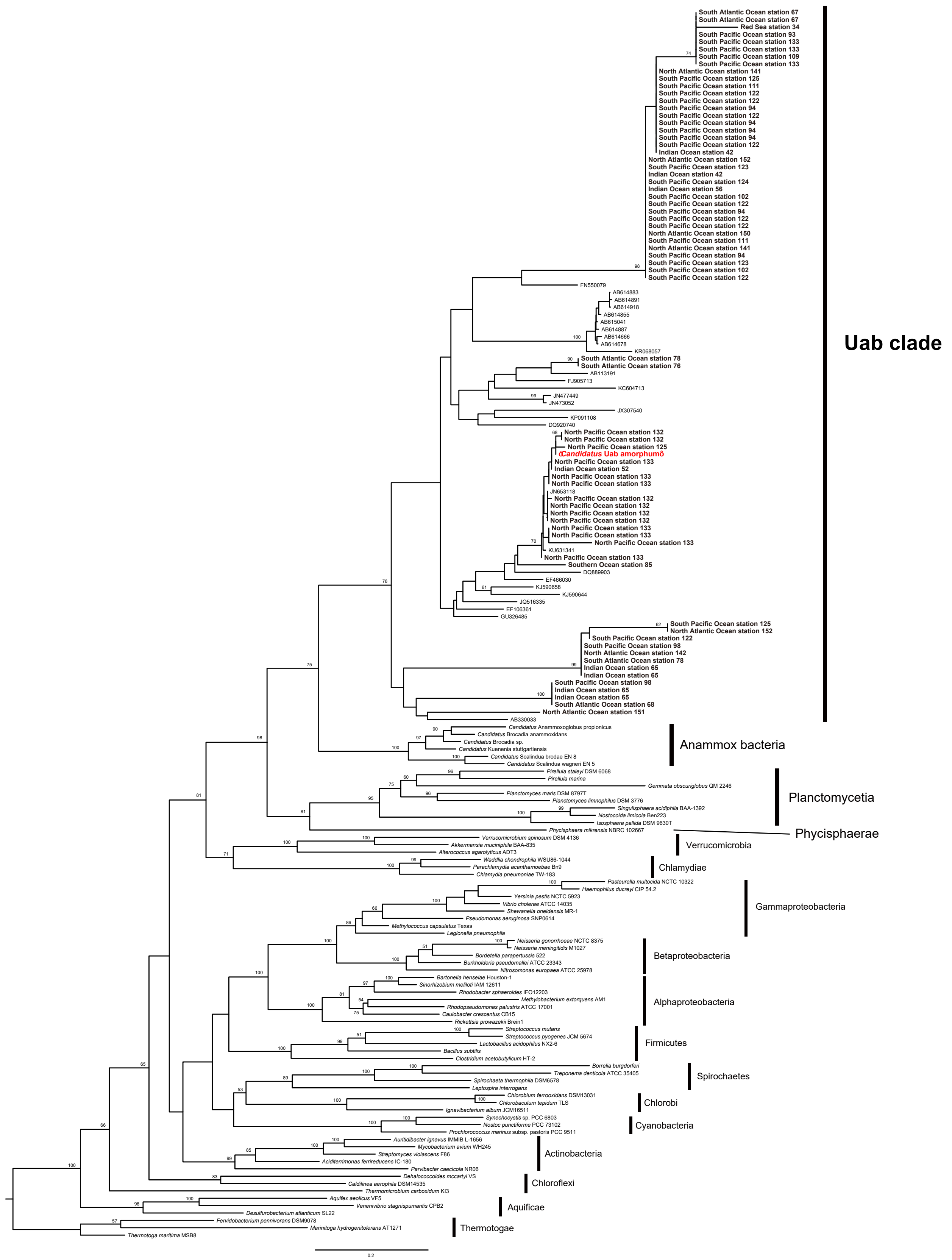

Supplementary Figure 5 | Maximum likelihood tree of 16S rRNA gene sequences including environmental sequences close to ó*Candidatus* Uab amorphumô. The tree was inferred from 1,509 nucleotide positions of 167 taxa. Taxa in bold type show the sequences from Tara oceans expedition. Left and right values on nodes indicate bootstrap value and Bayesian posterior probability, respectively. Scale bar indicates nucleotide substitution rate per site.

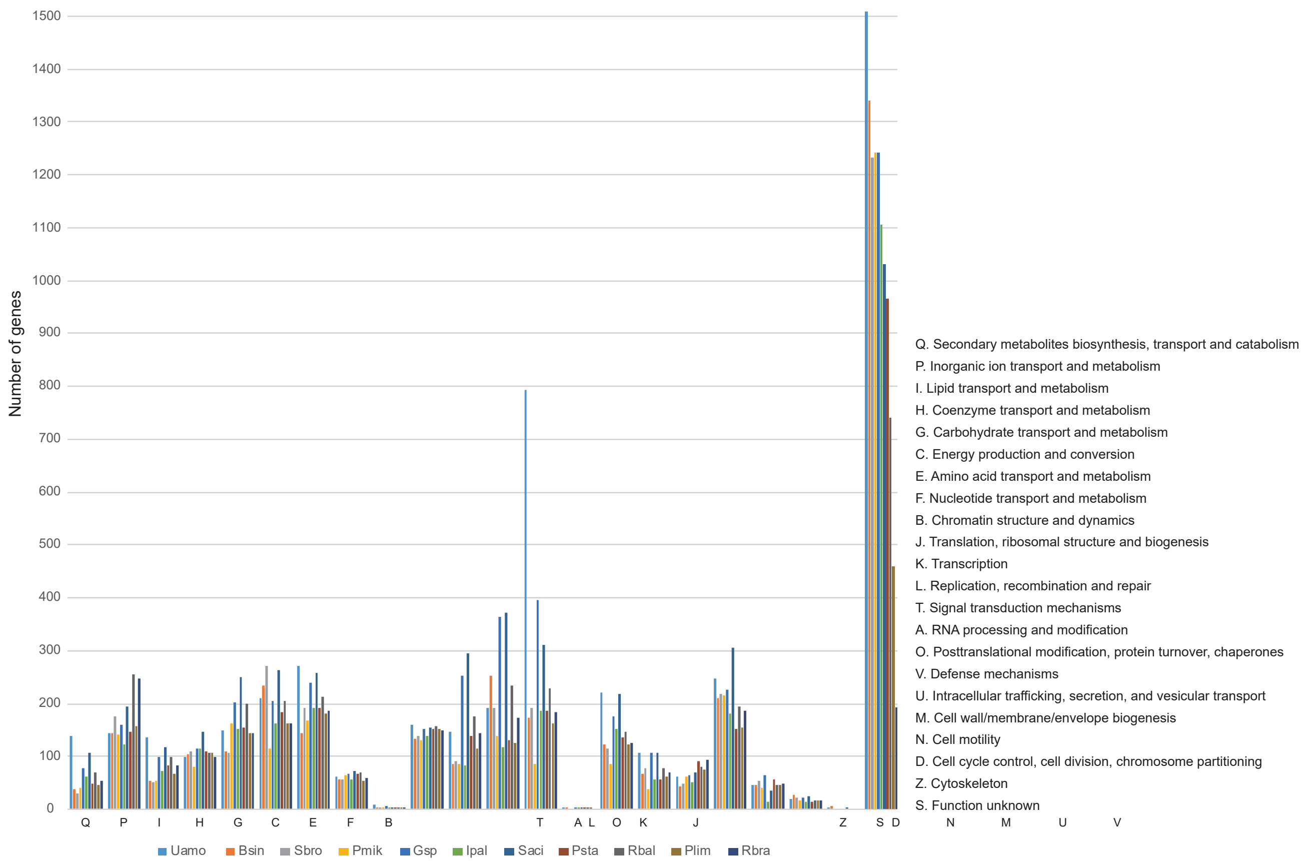

Supplementary Figure 6 | Comparison of COG category between ó*Candidatus* Uab amorphumô and nine planctomycetes. Uamo; ó*Ca.* Uab amorphumô. Bsin; ó*Candidatus* Brocadia sinicaô. Sbro; ó*Candidatus* Scalinduaô brodae. Pmik; *Phycisphaera mikurensis*. Gsp; *Gemmata* sp. Ipal; *Isosphaera pallida*. Saci; *Singulisphaera acidiphila*. Psta; *Pirellula staleyi*. Rbal; *Rhodopirellula baltica*. Plim; *Planctopirus limnophila*. Rbra; *Rubinisphaera brasiliensis*.

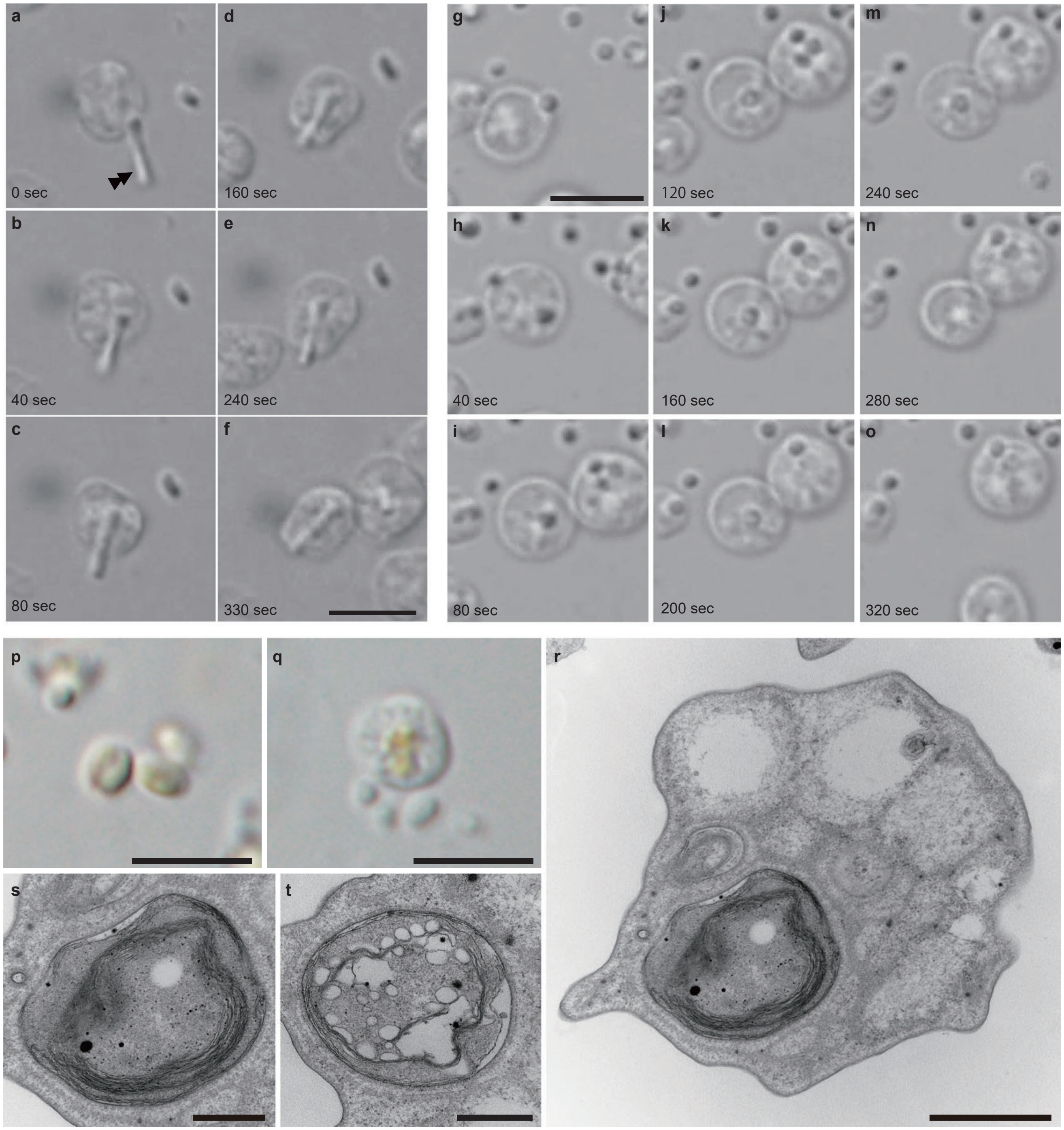

Supplementary Figure 7 | Light and electron micrographs of ó*Candidatus* Uab amorphumô that engulfs Gram-positive bacteria and eukaryote. (**a–f**) Selected images of time-lapse video showing that ó*Ca.* Uab amorphumô engulfs *Lactobacillus farciminis*. Double arrowhead indicate an engulfed cell of *Lactobacillus farciminis*. (**g–o**) Selected images of time-lapse video showing that ó*Ca*. Uab amorphumô engulfs *Staphylococcus condimenti*. Double arrowhead indicate an engulfed cell of *Staphylococcus condimenti* (**p**) Light micrograph of *Bathycoccus prasinos.* (**q**) Light micrograph of ó*Ca.* Uab amorphumô that engulfs *B. prasinos.* (**r**) Transmission electron micrograph of the phagosome-like vacuole (PV) of which includes intact *B. prasinos* cell. (**s**) PV including intact *B. prasinos* cell. (**t**) PV including disrupted *B. prasinos* cell. Scale bars, 5 µm (**a–q**),1 µm (**r**), and 500 nm (**s,t**).

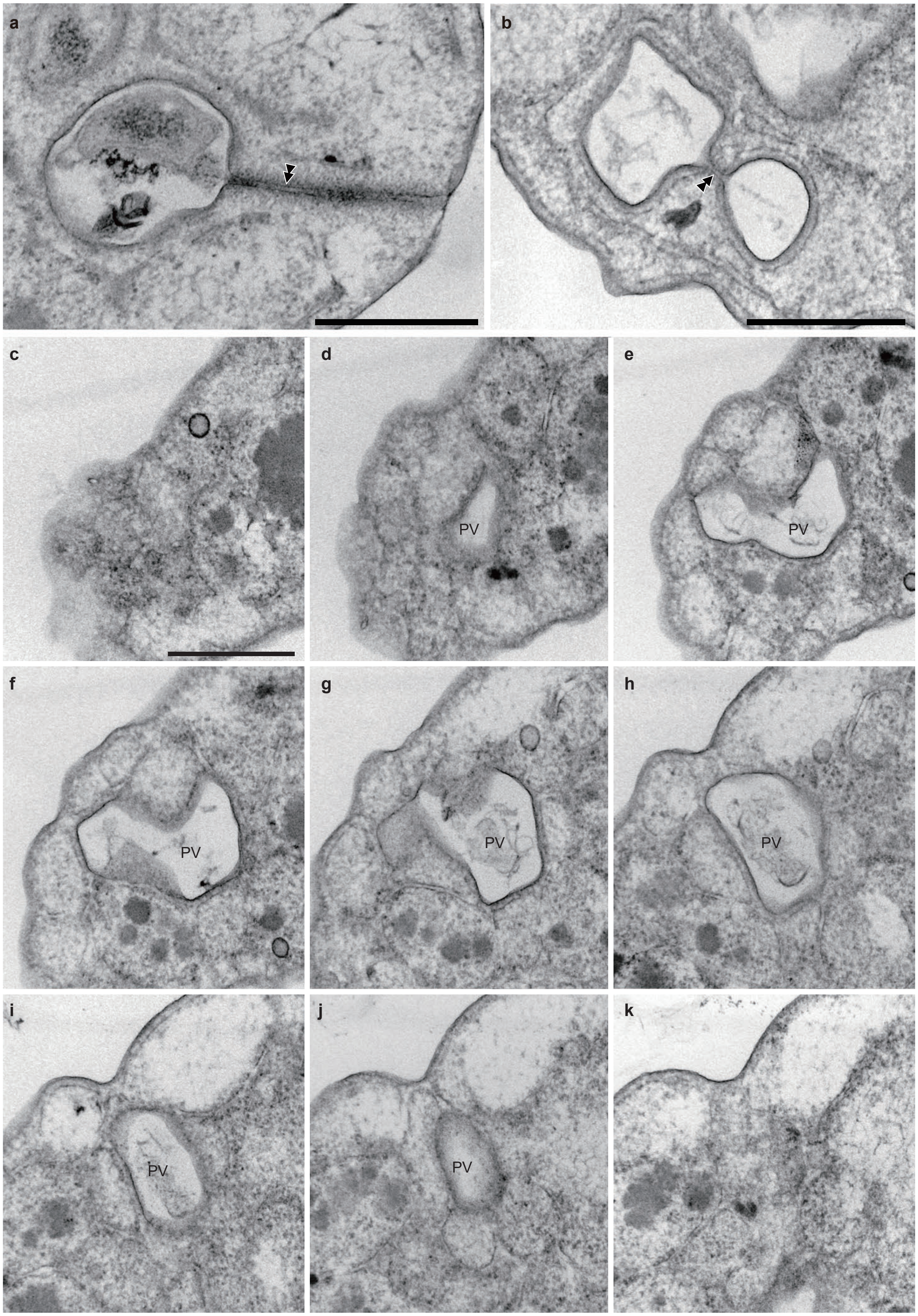

Supplementary Figure 8 | Transmission electron micrographs of phagosome-like vacuoles (PVs). (**a**) Duct (double arrowhead) connecting PV with outside of the cell. (b) Duct (double arrowhead) connecting two PVs. (**c–k**) Serial sections of 70 nm thickness showing a PV without duct. Scale bars, 500 nm (**a,b**) and 200 nm (**c–k**)

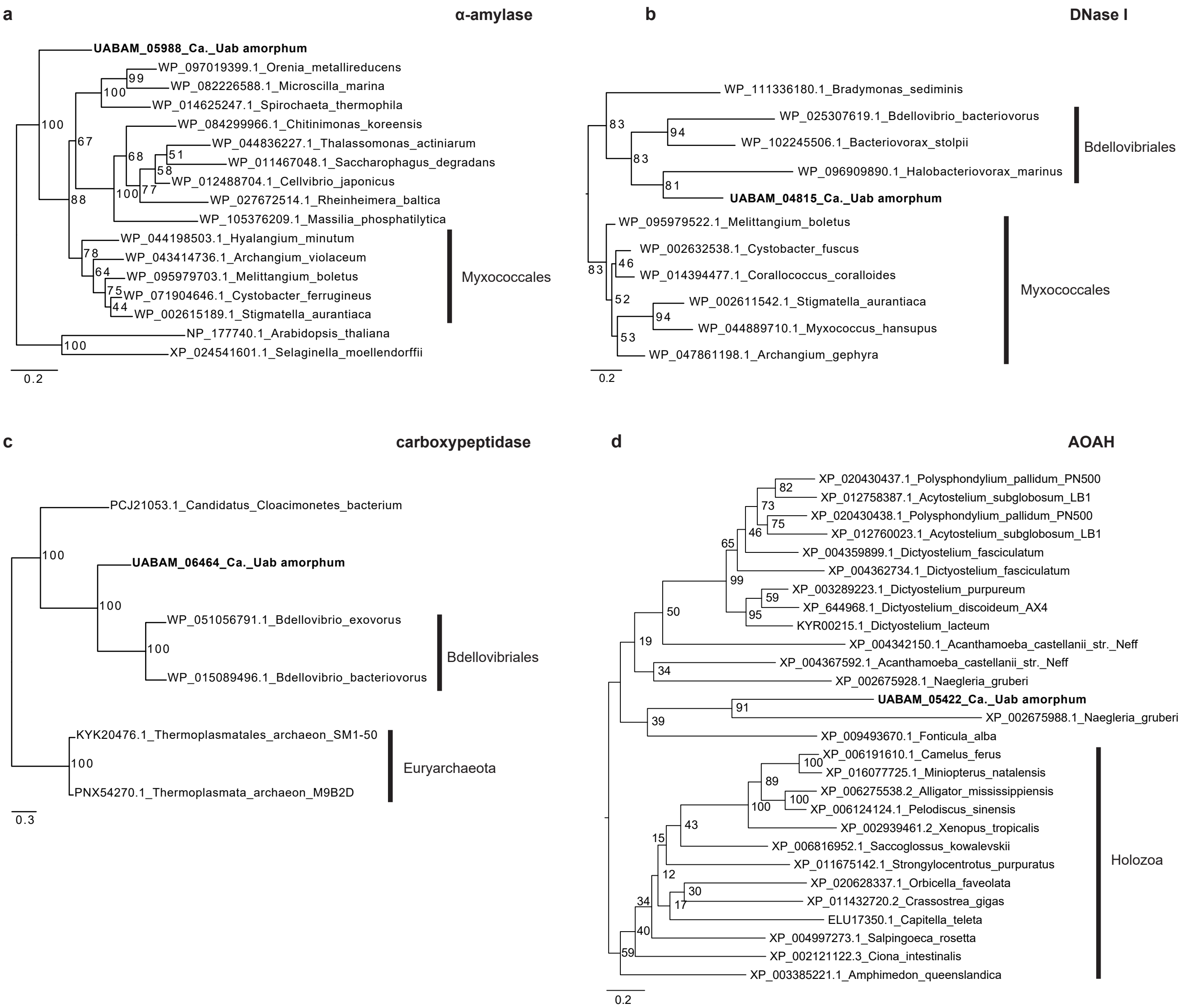

Supplementary Figure 9 | Maximum likelihood trees of digestive proteins putatively secreted or localized at the outer membrane. (**a**) Tree of α-amylase inferred from 331 amino acids of 17 OTUs. (**b**) Tree of DNase I inferred from 173 amino acids of 11 OTUs. (**c**) Tree of carboxypeptidase inferred from 206 amino acids of 6 OTUs. (**d**) Tree of acyloxyacyl hydrolase (AOAH) inferred from 534 amino acids of 28 OTUs. Bootstrap values are shown on nodes. Scale bars indicate nucleotide substitution rate per site.

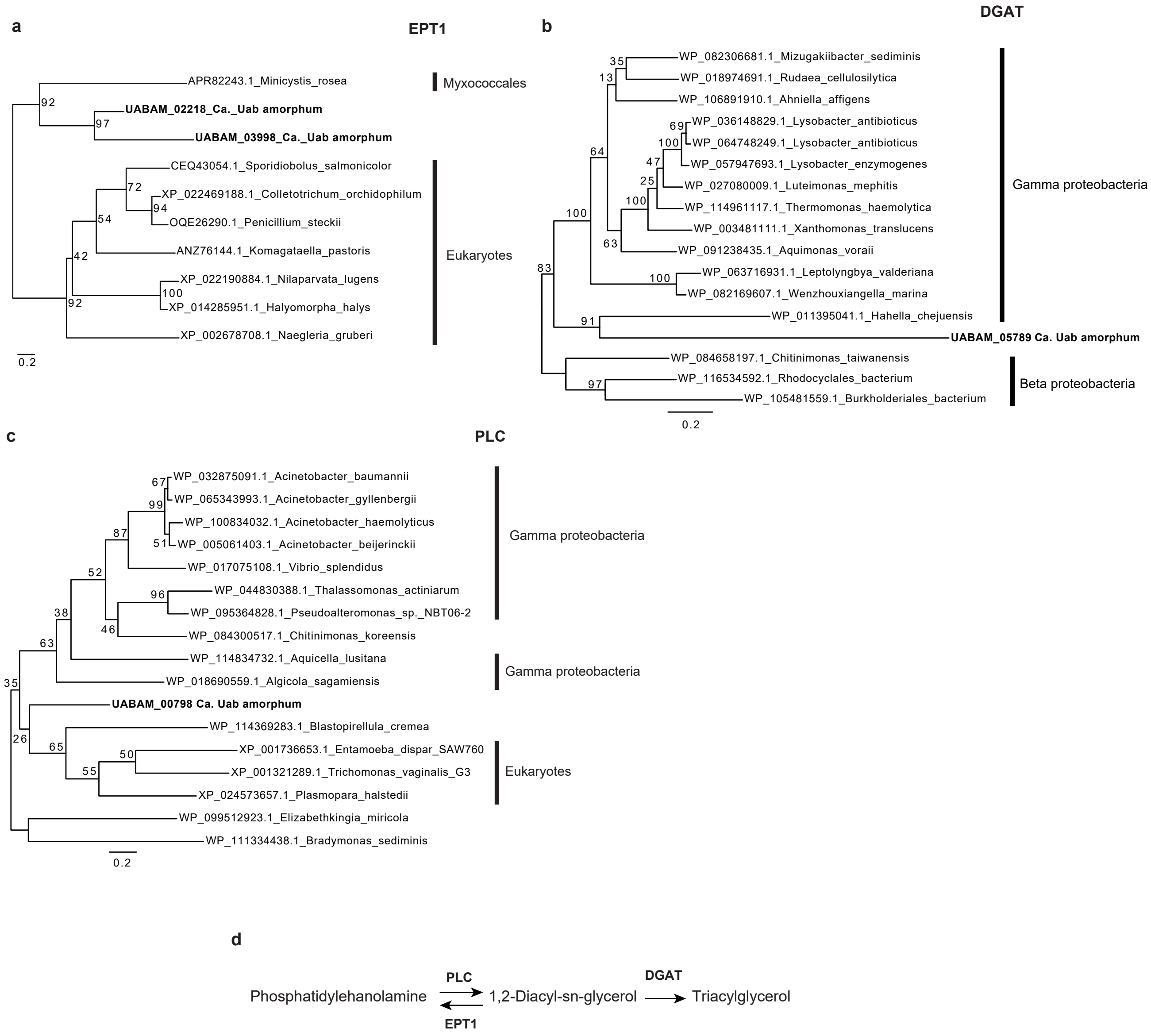

Supplementary Figure 10 | Maximum likelihood trees of proteins involved with lipid metabolism. (**a**) Tree of ethanolamine phosphotransferase (EPT1) inferred from 154 amino acids of 10 OTUs. (**b**) Tree of diacylglycerol acyltransferase (DGAT) inferred from 408 amino acids of 17 OTUs. (**c**) Tree of phospholipase c (PLC) inferred from 225 amino acids of 17 OTUs. Bootstrap values are shown on nodes. (**d**) Inferred metabolic pathway between phosphatidylethanolamine and triacylglycerol. Scale bars indicate nucleotide substitution rate per site.

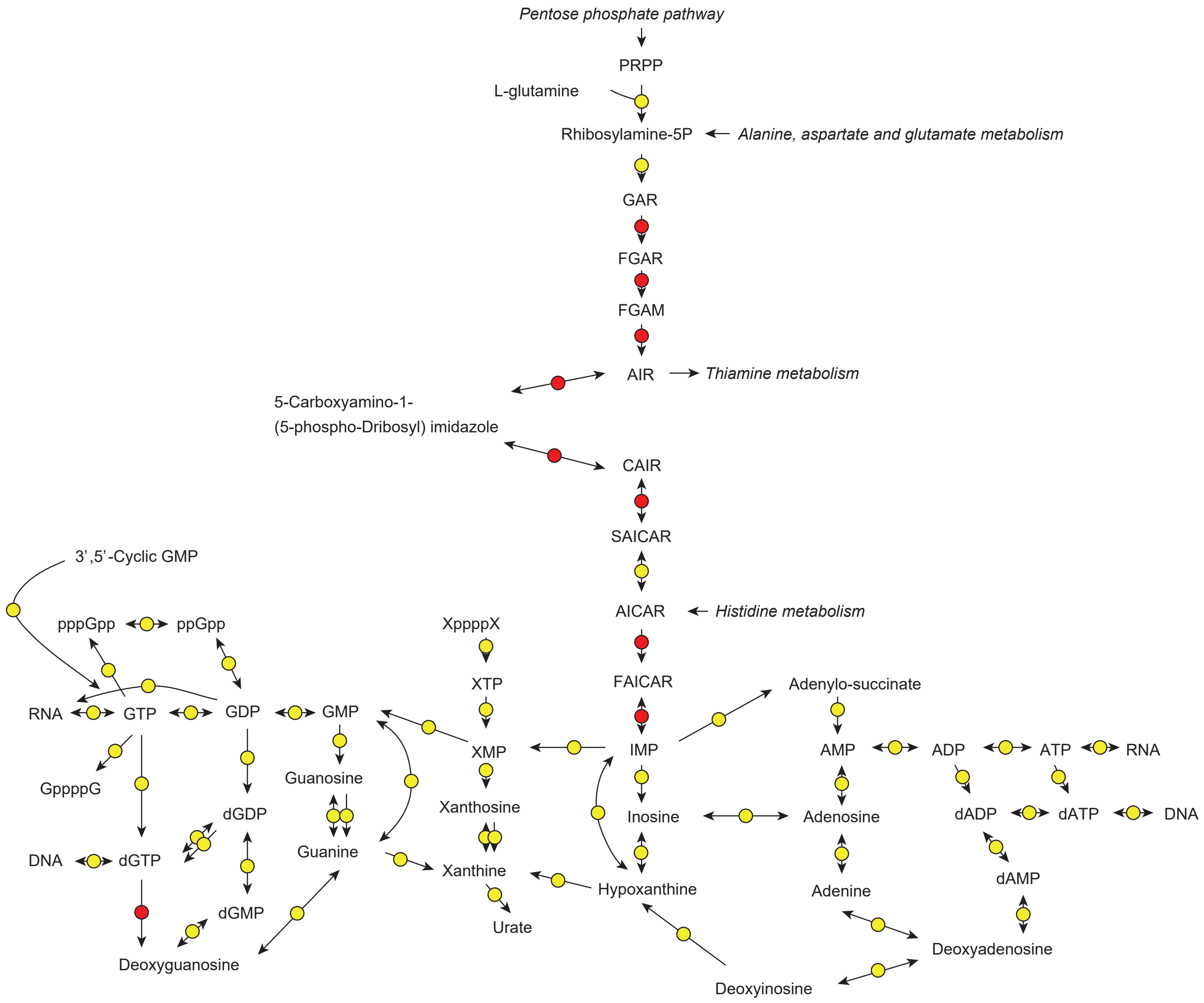

Supplementary Figure 11 | Biosynthesis pathway of purine nucleotides in '*Candidatus* Uab amorphum'. Red circles indicate genes absent in '*Ca*. Uab amorphum'. Yellow circles indicate genes present in '*Ca*. Uab amorphum'.

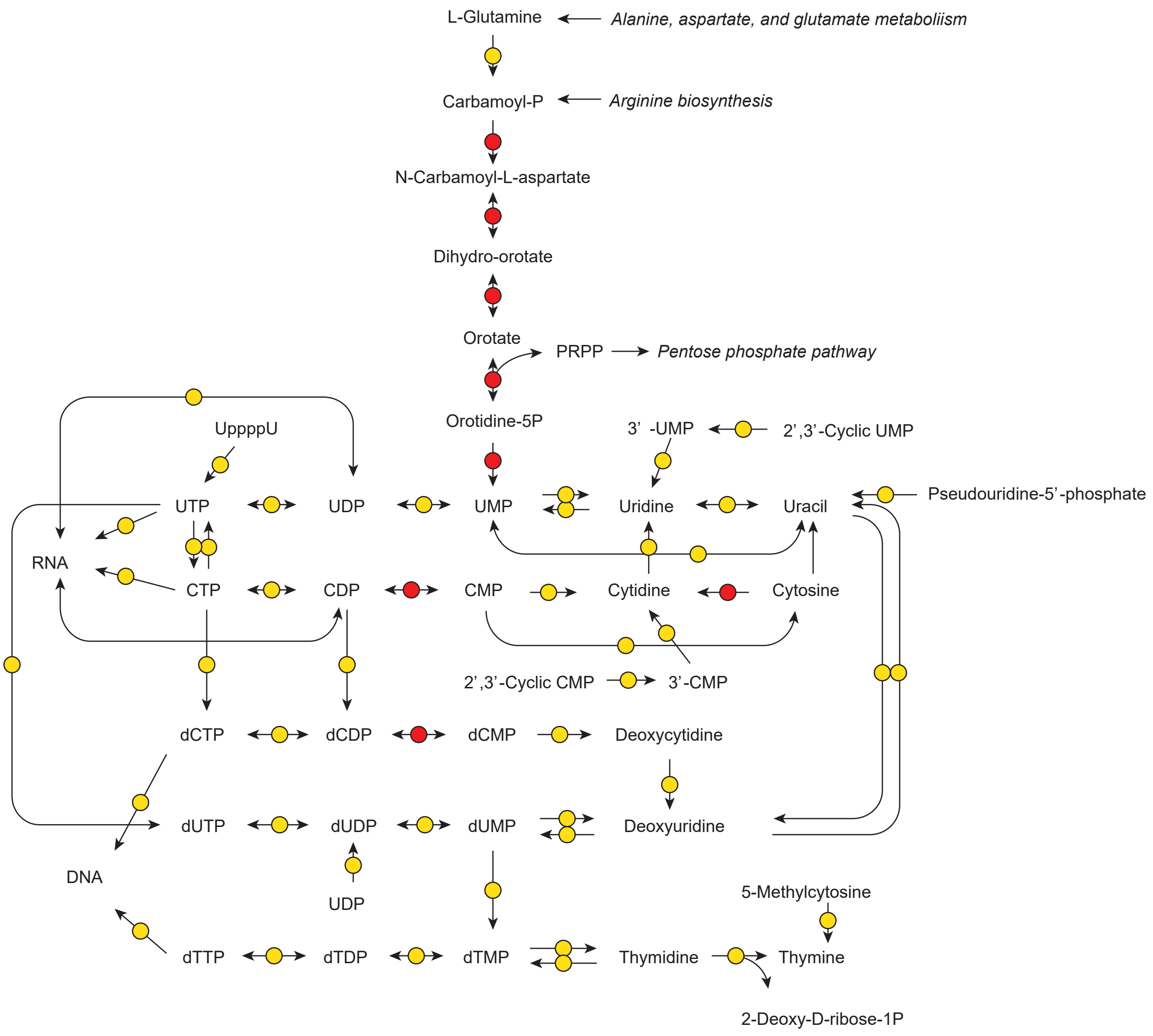

Supplementary Figure 12 | Biosynthesis pathway of pyrimidine nucleotides in '*Candidatus* Uab amorphum'. Red circles indicate genes absent in '*Ca*. Uab amorphum'. Yellow circles indicate genes present in '*Ca*. Uab amorphum'.

Supplementary References

1. Amann, R.I. et al. Combination of 16S rRNA-targeted oligonucleotide probes with flow cytometry for analyzing mixed microbial populations. *Appl. Environ. Microbiol.* **56**, 1919–1925 (1990).

2. Neef, A., Amann, R., Schlesner, H. & Schleifer, K.H. Monitoring a widespread bacterial group: in situ detection of planctomycetes with 16S rRNA-targeted probes. *Microbiology* **144**, 3257–3266 (1998).
