# Supplementary material for: Phagocytosis-like cell engulfment by a planctomycete bacterium
Source: Nat Commun. 2019 Dec 11;10:5529. doi: 10.1038/s41467-019-13499-2 (PMC6906331; doi:10.1038/s41467-019-13499-2)
Supplement: Supplementary file 2 — Description of Additional Supplementary Files [file 41467_2019_13499_MOESM2_ESM.pdf]

Description of Additional Supplementary Files

File Name: Supplementary Data 1
Description: Peptidases in Candidatus Uab amorphum genome.

File Name: Supplementary Data 2
Description: Genes related to amino acid biosynthesis.

File Name: Supplementary Movie 1
Description: Locomotion of *Candidatus* Uab amorphum, time-lapse (20×).

File Name: Supplementary Movie 2
Description: Phagocytosis-like cell engulfment of *Candidatus* Uab amorphum, time-lapse (20×).

File Name: Supplementary Movie 3
Description: *Candidatus* Uab amorphum and *Debaryomyces hansenii* showing *Ca.* Uab amorphum does not engulf *D. hansenii*, time-lapse (40×).

File Name: Supplementary Movie 4
Description: *Candidatus* Uab amorphum and AcGFP1-labelled *Escherichia coli* showing *Ca.* Uab amorphum engulf *E. coli*, time-lapse (600×).
