# Supplementary material for: Phagocytosis-like cell engulfment by a planctomycete bacterium
Source: Nat Commun. 2019 Dec 11;10:5529. doi: 10.1038/s41467-019-13499-2 (PMC6906331; doi:10.1038/s41467-019-13499-2)
Supplement: Supplementary file 9 — Reporting Summary [file 41467_2019_13499_MOESM9_ESM.pdf]

nature research

Corresponding author(s): Takashi Shiratori

Last updated by author(s): Jul 11, 2019

Reporting Summary

Nature Research wishes to improve the reproducibility of the work that we publish. This form provides structure for consistency and transparency in reporting. For further information on Nature Research policies, see Authors & Referees and the Editorial Policy Checklist.

Statistics

For all statistical analyses, confirm that the following items are present in the figure legend, table legend, main text, or Methods section.

n/a	Confirmed	
☒	☐	The exact sample size (*n*) for each experimental group/condition, given as a discrete number and unit of measurement
☒	☐	A statement on whether measurements were taken from distinct samples or whether the same sample was measured repeatedly
☒	☐	The statistical test(s) used AND whether they are one- or two-sided<br>*Only common tests should be described solely by name; describe more complex techniques in the Methods section.*
☒	☐	A description of all covariates tested
☒	☐	A description of any assumptions or corrections, such as tests of normality and adjustment for multiple comparisons
☒	☐	A full description of the statistical parameters including central tendency (e.g. means) or other basic estimates (e.g. regression coefficient) AND variation (e.g. standard deviation) or associated estimates of uncertainty (e.g. confidence intervals)
☒	☐	For null hypothesis testing, the test statistic (e.g. *F*, *t*, *r*) with confidence intervals, effect sizes, degrees of freedom and *P* value noted<br>*Give P values as exact values whenever suitable.*
☐	☒	For Bayesian analysis, information on the choice of priors and Markov chain Monte Carlo settings
☒	☐	For hierarchical and complex designs, identification of the appropriate level for tests and full reporting of outcomes
☒	☐	Estimates of effect sizes (e.g. Cohen's *d*, Pearson's *r*), indicating how they were calculated

Our web collection on statistics for biologists contains articles on many of the points above.

Software and code

Policy information about availability of computer code

Data collection	no software was used for data collection
Data analysis	CodonCode Aligner version 7, MAFFT v7.221, MAFFT v7.273 SeaView version 4.6, jModelTest 2.1.10, RAxML version 8.1.15, MrBayes 3.2.2, MrBayes 3.2.6, Canu v1.4, Pbjelly 0.3.0, Quiver 2.1.0, , IQ-TREE 1.4.3, IQ-TREE 1.5.5, PSORTb 3.0, Cello v.2.5, HGTector v0.2.1, MEGA 7

For manuscripts utilizing custom algorithms or software that are central to the research but not yet described in published literature, software must be made available to editors/reviewers. We strongly encourage code deposition in a community repository (e.g. GitHub). See the Nature Research guidelines for submitting code & software for further information.

Data

Policy information about availability of data

All manuscripts must include a data availability statement. This statement should provide the following information, where applicable:

- Accession codes, unique identifiers, or web links for publicly available datasets
- A list of figures that have associated raw data
- A description of any restrictions on data availability

The monoxenic culture of Ca. Uab amorphum was deposited in Japan Collection of Microorganisms (JCM) as JCM xxxx and in American Type Culture Collection (ATCC) as ATCC xxxx. The 16S rRNA gene sequence of Ca. Uab amorphum was deposited in DDBJ/GenBank/ENA with the accession number LC496071 [https://www.ncbi.nlm.nih.gov/nuccore/LC496071.1/]. The genome assembly was deposited in DDBJ/GenBank/ENA with accession numbers AP019860 [https://www.ncbi.nlm.nih.gov/nuccore/AP019860.1/].

Field-specific reporting

Please select the one below that is the best fit for your research. If you are not sure, read the appropriate sections before making your selection.

☒ Life sciences ☐ Behavioural & social sciences ☐ Ecological, evolutionary & environmental sciences

For a reference copy of the document with all sections, see nature.com/documents/nr-reporting-summary-flat.pdf

Life sciences study design

All studies must disclose on these points even when the disclosure is negative.

Sample size	N/A
Data exclusions	No data were excluded from the analyses
Replication	N/A
Randomization	N/A
Blinding	N/A

Reporting for specific materials, systems and methods

We require information from authors about some types of materials, experimental systems and methods used in many studies. Here, indicate whether each material, system or method listed is relevant to your study. If you are not sure if a list item applies to your research, read the appropriate section before selecting a response.

Materials & experimental systems

n/a	Involved in the study	
☒	☐	Antibodies
☒	☐	Eukaryotic cell lines
☒	☐	Palaeontology
☐	☒	Animals and other organisms
☒	☐	Human research participants
☒	☐	Clinical data

Methods

n/a	Involved in the study	
☒	☐	ChIP-seq
☒	☐	Flow cytometry
☒	☐	MRI-based neuroimaging

Animals and other organisms

Policy information about studies involving animals; ARRIVE guidelines recommended for reporting animal research

Laboratory animals	This study did not involve laboratory animals
Wild animals	This study did not involve wild animals
Field-collected samples	This study did not involve samples collected from the field
Ethics oversight	This study did not use animals. This study does not involve bioweapons.

Note that full information on the approval of the study protocol must also be provided in the manuscript.
